# The Art of Happiness: An Explorative Study of a Contemplative Program for Subjective Well-Being

**DOI:** 10.3389/fpsyg.2021.600982

**Published:** 2021-02-11

**Authors:** Clara Rastelli, Lucia Calabrese, Constance Miller, Antonino Raffone, Nicola De Pisapia

**Affiliations:** ^1^Department of Psychology and Cognitive Science, University of Trento, Trento, Italy; ^2^Department of Psychology, Sapienza University of Rome, Rome, Italy; ^3^Institute Lama Tzong Khapa, Pisa, Italy

**Keywords:** meditation, wisdom, happiness, well–being, mindfulness

## Abstract

In recent decades, psychological research on the effects of mindfulness-based interventions has greatly developed and demonstrated a range of beneficial outcomes in a variety of populations and contexts. Yet, the question of how to foster subjective well-being and happiness remains open. Here, we assessed the effectiveness of an integrated mental training program *The Art of Happiness* on psychological well-being in a general population. The mental training program was designed to help practitioners develop new ways to nurture their own happiness. This was achieved by seven modules aimed at cultivating positive cognition strategies and behaviors using both formal (i.e., lectures, meditations) and informal practices (i.e., open discussions). The program was conducted over a period of 9 months, also comprising two retreats, one in the middle and one at the end of the course. By using a set of established psychometric tools, we assessed the effects of such a mental training program on several psychological well-being dimensions, taking into account both the longitudinal effects of the course and the short-term effects arising from the intensive retreat experiences. The results showed that several psychological well-being measures gradually increased within participants from the beginning to the end of the course. This was especially true for life satisfaction, self-awareness, and emotional regulation, highlighting both short-term and longitudinal effects of the program. In conclusion, these findings suggest the potential of the mental training program, such as *The Art of Happiness*, for psychological well-being.

## Introduction

People desire many valuable things in their life, but—more than anything else—they want happiness (Diener, 2000). The sense of happiness has been conceptualized as people's experienced well-being in both thoughts and feelings (Diener, 2000; Kahneman and Krueger, 2006). Indeed, research on well-being suggests that the resources valued by society, such as mental health (Koivumaa-Honkanen et al., 2004) and a long life (Danner et al., 2001), associate with high happiness levels. Since the earliest studies, subjective well-being has been defined as the way in which individuals experience the quality of their life in three different but interrelated mental aspects: infrequent negative affect, frequent positive affect, and cognitive evaluations of life satisfaction in various domains (physical health, relationships, and work) (Diener, 1984, 1994, 2000; Argyle et al., 1999; Diener et al., 1999; Lyubomksky et al., 2005; Pressman and Cohen, 2005). A growing body of research has been carried out aimed at identifying the factors that affect happiness, operationalized as subjective well-being. In particular, the construct of happiness is mainly studied within the research fields of positive psychology or contemplative practices, which are grounded in ancient wisdom traditions. Positive psychology has been defined as the “the scientific study of human strengths and virtues” (Sheldon and King, 2001), and it can be traced back to the reflections of Aristotle about different perspectives on well-being (Ryan and Deci, 2001). On the other end, contemplative practices include a great variety of mental exercises, such as mindfulness, which has been conceived as a form of awareness that emerges from experiencing the present moment without judging those experiences (Kabat-Zinn, 2003; Bishop et al., 2004). Most of these exercises stem from different Buddhist contemplative traditions such as Vipassana and Mahayana (Kornfield, 2012). Notably, both perspective share the idea of overcoming suffering and achieving happiness (Seligman, 2002). Particularly, Buddhism supports “the cultivation of happiness, genuine inner transformation, deliberately selecting and focusing on positive mental states” (Lama and Cutler, 2008). In addition, mindfulness has been shown to be positively related to happiness (Shultz and Ryan, 2015), contributing to eudemonic and hedonic well-being (Howell et al., 2011).

In fact, although the definition of happiness has a long history and goes back to philosophical arguments and the search for practical wisdom, in modern times, happiness has been equated with hedonism. It relies on the achievement of immediate pleasure, on the absence of negative affect, and on a high degree of satisfaction with one's life (Argyle et al., 1999). Nonetheless, scholars now argue that authentic subjective well-being goes beyond this limited view and support an interpretation of happiness as a eudemonic endeavor (Ryff, 1989; Keyes, 2006; Seligman, 2011; Hone et al., 2014). Within this view, individuals seem to focus more on optimal psychological functioning, living a deeply satisfying life and actualizing their own potential, personal growth, and a sense of autonomy (Deci and Ryan, 2008; Ryff, 2013; Vazquez and Hervas, 2013; Ivtzan et al., 2016). In psychology, such a view finds one of its primary supports in Maslow's (1981) theory of human motivation. Maslow argued that experience of a higher degree of satisfaction derives from a more wholesome life conduct. In Maslow's hierarchy of needs theory, once lower and more localized needs are satisfied, the unlimited gratification of needs at the highest level brings people to a full and deep experience of happiness (Inglehart et al., 2008). Consequently, today, several scholars argue that high levels of subjective well-being depend on a multi-dimensional perspective, which encompasses both hedonic and eudemonic components (Huta and Ryan, 2010; Ryff and Boylan, 2016). Under a wider perspective, the process of developing well-being reflects the notion that mental health and good functioning are more than a lack of illness (Keyes, 2005). This approach is especially evident if we consider that even the definition of mental health has been re-defined by the World Health Organization (1948), which conceives health not merely as the absence of illness, but as a whole state of biological, psychological, and social well-being.

To date, evidence exists suggesting that happiness is, in some extent, modulable and trainable. Thus, simple cognitive and behavioral strategies that individuals choose in their lives could enhance happiness (Lyubomirsky et al., 2005; Sin and Lyubomirsky, 2009). In the history of psychology, a multitude of clinical treatments have been applied to minimize the symptoms of a variety of conditions that might hamper people from being happy, such as anger, anxiety, and depression (for instance, see Forman et al., 2007; Spinhoven et al., 2017). In parallel with this view, an alternative—and less developed—perspective found in psychology focuses on the scientific study of individual experiences and positive traits, not for clinical ends, but instead for personal well-being and flourishing (e.g., Fredrickson and Losada, 2005; Sin and Lyubomirsky, 2009). Yet, the question of exactly how to foster subjective well-being and happiness, given its complexity and importance, remains open to research. Answering this question is of course of pivotal importance, both individually and at the societal level. Positive Psychology Interventions encompass simple, self-administered cognitive behavioral strategies intended to reflect the beliefs and behaviors of individuals and, in response to that, to increase the happiness of the people practicing them (Sin and Lyubomirsky, 2009; Hone et al., 2015). Specifically, a series of comprehensive psychological programs to boost happiness exist, such as Fordyce's program (Fordyce, 1977), Well-Being Therapy (Fava, 1999), and Quality of Life Therapy (Frisch, 2006). Similarly, a variety of meditation-based programs aim to develop mindfulness and emotional regulatory skills (Carmody and Baer, 2008; Fredrickson et al., 2008; Weytens et al., 2014), such as Mindfulness-Based Stress Reduction (MBSR; Kabat-Zinn, 1990) and Mindfulness-Based Cognitive Therapy (MBCT; Teasdale et al., 2000). Far from being a mere trend (De Pisapia and Grecucci, 2017), those mindfulness-based interventions have been shown to lead to increased well-being (Baer et al., 2006; Keng et al., 2011; Choi et al., 2012; Coo and Salanova, 2018; Lambert et al., 2019) in several domains, such as cognition, consciousness, self, and affective processing (Raffone and Srinivasan, 2017). Typically, mindfulness programs consist of informal and formal practice that educate attention and develop one's capacity to respond to unpredicted and/or negative thoughts and experiences (Segal and Teasdale, 2002). In this context, individuals are gradually introduced to meditation practices, focusing first on the body and their own breath, and later on thoughts and mental states. The effects of these programs encompass positive emotions and reappraisal (Fredrickson et al., 2008; Grecucci et al., 2015; Calabrese and Raffone, 2017) and satisfaction in life (Fredrickson et al., 2008; Kong et al., 2014) and are related to a reduction of emotional reactivity to negative affect, stress (Arch and Craske, 2006; Jha et al., 2017), and aggressive behavior (Fix and Fix, 2013). All these effects mediate the relationship between meditation frequency and happiness (Campos et al., 2016). This allows positive psychology interventions to improve subjective well-being and happiness and also reduce depressive symptoms and negative affect along with other psychopathologies (Seligman, 2002; Quoidbach et al., 2015). Engaging in mindfulness might enhance in participants the awareness of what is valuable to them (Shultz and Ryan, 2015). This aspect has been related to the growth of self-efficacy and autonomous functioning and is attributable to an enhancement in eudemonic well-being (Deci and Ryan, 1980). Moreover, being aware of the present moment provides a clearer vision of the existing experience, which in turn has been associated with increases in hedonic well-being (Coo and Salanova, 2018). Following these approaches, recent research provides evidence that trainings that encompass both hedonic and eudemonic well-being are correlated with tangible improved health outcomes (Sin and Lyubomirsky, 2009).

Although there is a consistent interest in scientific research on the general topic of happiness, such studies present several limitations. Firstly, most of the research has focused on clinical studies to assess the effectiveness of happiness-based interventions—in line with more traditional psychological research, which is primarily concerned with the study of mental disorders (Garland et al., 2015, 2017; Groves, 2016). Secondly, most of the existing interventions are narrowly focused on the observation of single dimensions (i.e., expressing gratitude or developing emotional regulation skills) (Boehm et al., 2011; Weytens et al., 2014). Moreover, typically studies involve brief 1- to 2-week interventions (Gander et al., 2016), in contrast with the view that eudemonia is related to deep and long-lasting aspects of one's personal lifestyle. Furthermore, while the effectiveness of mindfulness-based therapies is well-documented, research that investigates the effects of mindfulness retreats has been lacking, which are characterized by the involvement of more intense practice from days to even years [for meta-analysis and review, see Khoury et al. (2017), McClintock et al. (2019), Howarth et al. (2019)].

In this article, we report the effects on subjective well-being of an integrated mental training program called *The Art of Happiness*, which was developed and taught by two of the authors (CM for the core course subject matter and NDP for the scientific presentations). The course lasted 9 months and included three different modules (see Methods and Supplementary Material for all details), namely, seven weekends (from Friday evening to Sunday afternoon) dedicated to a wide range of specific topics, two 5-day long retreats, and several free activities at home during the entire period. The course was designed to help practitioners develop new ways to nurture their own happiness, cultivating both self-awareness and their openness to others, thereby fostering their own emotional and social well-being. The basic idea was to let students discover how the union of ancient wisdom and spiritual practices with scientific discoveries from current neuropsychological research can be applied beneficially to their daily lives. This approach and mental training program was inspired by a book of the Fourteenth Dalai Lama Tenzin Gyatso and the psychiatrist Lama and Cutler (2008). The program rests on the principle that happiness is inextricably linked to the development of inner equilibrium, a kinder and more open perspective of self, others, and the world, with a key role given to several types of meditation practices. Additionally, happiness is viewed as linked to a conceptual understanding of the human mind and brain, as well as their limitations and potentiality, in the light of the most recent scientific discoveries. To this end, several scientific topics and discoveries from neuropsychology were addressed in the program, with a particular focus on cognitive, affective, and social neuroscience. Topics were taught and discussed with language suitable for the general public, in line with several recent books (e.g., Hanson and Mendius, 2011; Dorjee, 2013; Goleman and Davidson, 2017). The aim of this study was to examine how several psychological measures, related to psychological well-being, changed among participants in parallel with course attendance and meditation practices. Given the abovementioned results of the positive effects on well-being (Baer et al., 2006; Fredrickson et al., 2008; Keng et al., 2011; Choi et al., 2012; Kong et al., 2014; Coo and Salanova, 2018; Lambert et al., 2019), we predicted to find a significant increase in the dimensions of life satisfaction, control of anger, and mindfulness abilities. Conversely, we expected to observe a reduction of negative emotions and mental states (Arch and Craske, 2006; Fix and Fix, 2013; Jha et al., 2017)—i.e., stress, anxiety and anger. Moreover, our aim was to explore how those measures changed during the course of the mental training program, considering not only the general effects of the course (longitudinal effects) but also specific effects within each retreat (short-term effects). Our expectation for this study was therefore that the retreats would have had an effect on the psychological dimensions of well-being linked to the emotional *states* of our participants, while the whole course would have had a greater effect on the *traits* related to well-being. The conceptual distinction between states and traits was initially introduced in regard to anxiety by Cattell and Scheier (1961), and then subsequently further elaborated by Spielberger et al. (1983). When considering a mental construct (e.g., anxiety or anger), we refer to trait as a relatively stable feature, a general behavioral attitude, which reflects the way in which a person tends to perceive stimuli and environmental situations in the long term (Spielberger et al., 1983; Spielberger, 2010). For example, subjects with high trait anxiety have indeed anxiety as a habitual way of responding to stimuli and situations. The state, on the other hand, can be defined as a temporary phase within the emotional continuum, which, for example, in anxiety is expressed through a subjective sensation of tension, apprehension, and nervousness, and is associated with activation of the autonomic nervous system in the short term (Spielberger et al., 1983; Saviola et al., 2020). Here, in the adopted tests and analyses, we keep the two time scales separated, and we investigate the results with the aim of understanding the effects of the program on states and traits of different emotional and well-being measures. As a first effect of the course, we expect that the retreats affect mostly psychological states (as measured in the comparison of psychological variables between start and end of each retreat), whereas the full course is predicted to affect mainly psychological traits (as measured in the comparison of the psychological variables between start, middle, and end of the entire 9-month period).

## Materials and Methods

### Participants

The participants in the mental training program and in the related research were recruited from the Institute Lama Tzong Khapa (Pomaia, Italy) in a 9-month longitudinal study (seven modules and two retreats) on the effects of a program called *The Art of Happiness* (see Supplementary Material for full details of the program). Twenty-nine participants followed the entire program (there were nine dropouts after the first module). Their mean age was 52.86 years (range = 39–66; SD = 7.61); 72% were female. Participants described themselves as Caucasian, reaching a medium-high scholarly level with 59% of the participants holding an academic degree and 41% holding a high school degree. The participants were not randomly selected, as they were volunteers in the program. Most of them had no serious prior experience of meditation, only basic experience consisting of personal readings or watching video courses on the web, which overall we considered of no impact to the study. The only exclusion criteria were absence of a history of psychiatric or neurological disease, and not being currently on psychoactive medications. The study was approved by the Ethics Committee of the Sapienza University of Rome, and all participants gave written informed consent. The participants did not receive any compensation for participation in the study.

### Design

The overall effectiveness of the 9-month training was examined using a within-subjects design, with perceived stress, mindfulness abilities, etc. (Time: pre–mid–end) as the dependent variable. The effectiveness of the retreats was examined using a 2 × 2 factor within-subjects design (condition: pre vs. post; retreat: 1 vs. 2), with the same dependent variables. The specific contemplative techniques that were applied in the program are described in the Supplementary Material, the procedure is described in the *Procedure* section, and the measurements are described in the *Materials* section.

### Mental Training Program

The program was developed and offered at the Institute Lama Tzong Khapa (Pomaia, Italy). It was one of several courses that are part of the Institute's ongoing programs under the umbrella of “Secular Ethics and Universal Values.” These various programs provide participants with opportunities to discover how the interaction of ancient wisdom and spiritual practices with contemporary knowledge from current scientific research in neuropsychology can be applied extensively and beneficially to improve the quality of their daily lives.

Specifically, *The Art of Happiness* was a 9-month program, with one program activity each month, either a weekend module or a retreat; there were two retreats—a mid-course retreat and a concluding retreat (for full details on the program, see Supplementary Material). Each thematic module provided an opportunity to sequentially explore the topics presented in the core course text, *The Art of Happiness* by the Lama and Cutler (2008).

In terms of the content of this program, as mentioned above, the material presented and explored has been drawn on the one hand from the teachings of Mahayana Buddhism and Western contemplative traditions, and current scientific research found in neuropsychology on the other hand. On the scientific side, topics included the effects of mental training and meditation, the psychology and neuroscience of well-being and happiness, neuroplasticity, mind–brain–body interactions, different areas of contemplative sciences, the placebo effects, the brain circuits of attention and mind wandering, stress and anxiety, pain and pleasure, positive and negative emotions, desire and addiction, the sense of self, empathy, and compassion (for a full list of the scientific topics, see Supplementary Material).

The overall approach of the course was one of non-dogmatic exploration. Topics were presented not as undisputed truths, but instead as information to be shared, explored, examined, and possibly verified by one's own experience. Participants were heartily invited to doubt, explore, and test everything that was shared with them, to examine and experience firsthand whether what was being offered has validity or not.

The course was, essentially, an informed and gentle training of the mind, and in particular of emotions, based on the principle that individual well-being is inextricably linked to the development of inner human virtues and strengths, such as emotional balance, inner self-awareness, an open and caring attitude toward self and others, and clarity of mind that can foster a deeper understanding of one's own and others' reality.

The program provided lectures and discussions, readings, and expert videos introducing the material pertinent to each module's topic. Participants engaged with the material through listening, reading, discussing, and questioning. Participants were provided with additional learning opportunities to investigate each topic more deeply, critically, and personally, through the media of meditation, journaling, application to daily life, exercises at home, and contemplative group work with other participants in dyads and triads. Participants were then encouraged to reflect repeatedly on their insights and on their experiences, both successful and not, to apply their newly acquired understandings to their lives, by incorporating a daily reflection practice into their life schedule. The two program retreats also provided intensive contemplative experiences and activities, both individual and in dialogue with others.

On this basis, month after month in different dedicated modules, participants learned new ways to nurture their own happiness, to cultivate their openness to others, to develop their own emotional and social well-being, and to understand some of the scientific discoveries on these topics.

The specific topics addressed in corresponding modules and retreats, each in a different and consecutive month, were as follows: (1) The Purpose of Life: Authentic Happiness; (2) Empathy and Compassion; (3) Transforming Life's Suffering; (4) Working with Disturbing Emotions I: Hate and Anger; first retreat (intermediate); (5) Working with Disturbing Emotions II: The Self Image; (6) Life and Death; (7) Cultivating the Spiritual Dimension of Life: A Meaningful Life; second retreat (final). Full details of the entire program are reported in the Supplementary Material.

Participants were guided in the theory and practice of various contemplative exercises throughout the course pertaining to all the different themes. Recorded versions of all the various meditation exercises were made available to participants, enabling them to repeat these practices at home at their own pace.

Participants were encouraged to enter the program already having gained some basic experience of meditation, but this was not a strict requirement. In fact, not all participants in this experiment actually fulfilled this (only five), although each of the other participants had previous basic experiences of meditation (through personal readings, other video courses, etc.). In spite of this variety, by the end of the 9-month program, all participants were comfortable with contemplative practices in general and more specifically with the idea of maintaining a meditation practice in their daily lives.

During the various Art of Happiness modules, a variety of basic attentional and mindful awareness meditations were practiced in order to enhance attentional skills and cultivate various levels of cognitive, emotional, social, and environmental awareness.

Analytical and reflective contemplations are a form of deconstructive meditation (Dahl et al., 2015), which were applied during the course in different contexts. On the one hand, these types of meditations were applied in the context of heart-opening practices—for example, in the cultivation of gratitude, forgiveness, loving-kindness toward self and others, self-compassion, and compassion for others. Analytical and reflective meditations were also practiced as a learning tool for further familiarization with some of the more philosophical subject matter of the course—engaging in a contemplative analysis of impermanence (for example, contemplating more deeply and personally the transitory nature of one's own body, of one's own emotions and thoughts, as well as of the material phenomena that surround us). These analytical meditations were also accompanied by moments of concentration (sustained attention) at the conclusion of each meditation focusing on what the meditator has learned or understood in the meditative process, in order to stabilize and reinforce those insights more deeply within the individual.

Additional contemplative activities were also included in the program: contemplative art activities, mindful listening, mindful dialogue, and the practice of keeping silence during the retreat. Participants were, in addition, encouraged to keep a journal of their experiences during their Art of Happiness journey, especially in relation to their meditations and the insights and questions that emerged within themselves, in order to enhance their self-awareness and cultivate a deeper understanding of themselves, their inner life and well-being, and their own inner development during the course and afterward.

During the two retreats, the previous topics were explored again (modules 1–4 for the intermediary retreat and modules 5–7 for the final retreat), but without discussing the theoretical aspects (i.e., the neuroscientific and psychological theories), instead only focusing on the contemplative practices, which were practiced extensively for the whole day, both individually and in group activities (for a full list of the contemplative practices and retreat activities, see Supplementary Material).

### Procedure

**Figure 1:**
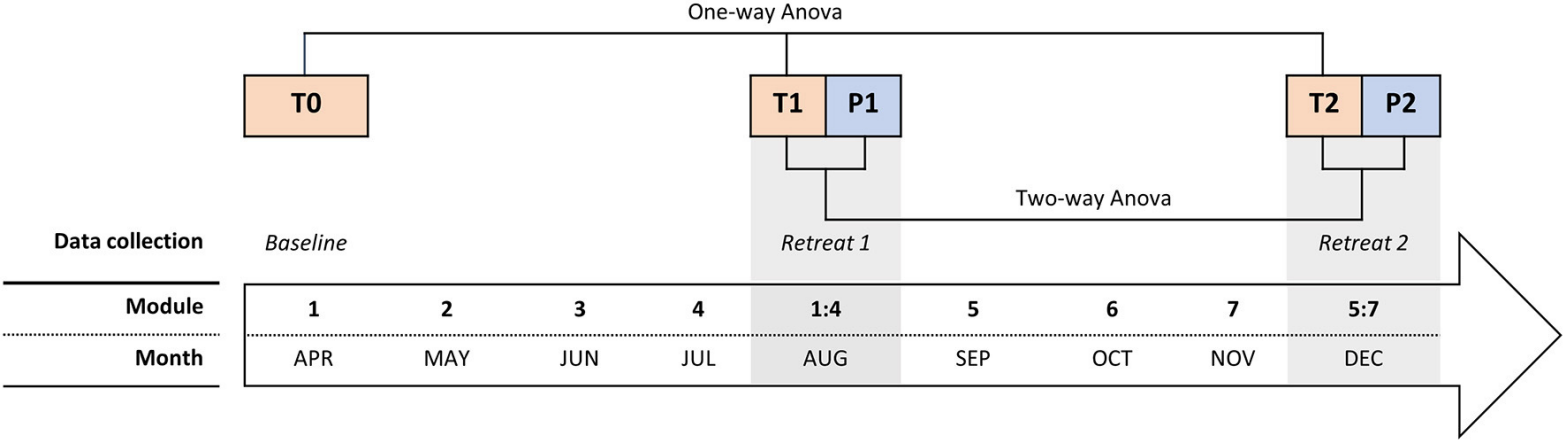
The timing of the course and the experimental procedure, including the modules, the retreats, and the 5 data collections (from T0 to P2).

We collected data at five-time points, always during the first day (either of the module or the retreat): at baseline (month 1 - T0), at pre (T1) and post (P1) of the mid-course retreat (month 5–Retreat 1), and at pre (T2) and post (R2) of the final retreat (month 9–Retreat 2), as shown in Figure 1. Participants filled out the questionnaires on paper all together within the rooms of the Institute Lama Tzong Khapa at the beginning of each module or retreat, and at the end of the retreats, with the presence of two researchers. The order of the questionnaires was randomized, per person and each questionnaire session lasted less than an hour.

### Materials

The adopted questionnaires were those commonly used in the literature to measure a variety of traits and states linked to well-being. An exhaustive description of the self-reported measures follows below.

#### Satisfaction With Life Scale (SWLS)

The SWLS (Diener et al., 1985) was developed to represent cognitive judgments of life satisfaction. Participants indicated their agreement in five items with a seven-point Likert scale, ranging from 1 (strongly disagree) to 7 (strongly agree). Scores range from 5 to 35, with higher scores representing higher levels of satisfaction. Internal consistency is very good with Cronbach's α = 0.85 [Italian version of the normative data in Di Fabio and Palazzeschi (2012)].

#### Short Version of the Perceived Stress Scale (PSS-10)

The PSS (Cohen et al., 1983) was designed to assess individual perception and reaction to stressful daily-life situations. The questionnaire consists of 10 questions related to the feelings and thoughts of the last month, with a value ranging from 0 (never) to 4 (very often) depending on the severity of the disturbance caused. Scores range from 0 to 40. Higher scores represent higher levels of perceived stress, reflecting the degree to which respondents find their lives unpredictable or overloaded. Cronbach's α ranges from 0.78 to 0.93 [Italian version of the normative data by Mondo et al. (2019)].

#### State-Trait Anxiety Inventory (STAI)

The STAI (Spielberger et al., 1983) was developed to assess anxiety. It has 40 items, on which respondents evaluate themselves in terms of frequency with a four-point Likert scale ranging from 1 (almost never) to 4 (almost always). The items are grouped in two independent subscales of 20 items each that assess state anxiety, with questions regarding the respondents' feelings at the time of administration, and trait anxiety, with questions that explore how the participant feels habitually. The scores range from 20 to 80. Higher scores reflect higher levels of anxiety. Internal consistency coefficients for the scale ranged from 0.86 to 0.95 [Italian version of the normative data by Spielberger et al. (2012)].

#### Positive and Negative Affect Schedule (PANAS)

PANAS (Watson et al., 1988) measures two distinct and independent dimensions: positive and negative affect. The questionnaire consists of 20 adjectives, 10 for the positive affect subscale and 10 for the negative affect scale. The positive affect subscale reflects the degree to which a person feels enthusiastic, active, and determined while the negative affect subscale refers to some unpleasant general states such as anger, guilt, and fear. The test presents a five-point Likert scale (1 = very slightly or not at all; 5 = extremely). The alpha reliabilities are acceptably high, ranging from 0.86 to 0.90 for positive affect and from 0.84 to 0.87 for negative affect [Italian version of the normative data by Terracciano et al. (2003)].

#### Five Facet Mindfulness Questionnaire (FFMQ)

The FFMQ (Baer et al., 2008) was developed to assess mindfulness facets through 39 items rated on a five-point Likert scale, ranging from 1 (never or very rarely true) to 5 (very often or always true). A total of five subscales are included: attention and observation of one's own thoughts, feelings, perceptions, and emotions (*Observe*); the ability to describe thoughts in words, feelings, perceptions, and emotions (*Describe*); act with awareness, with attention focused and sustained on a task or situation, without mind wandering (*Act-aware*); non-judgmental attitude toward the inner experience (*Non-Judge*); and the tendency to not react and not to reject inner experience (*Non-React*). Normative data of the FFMQ have demonstrated good internal consistency, with Cronbach's α ranging from 0.79 to 0.87 [Italian version by Giovannini et al. (2014)].

#### State-Trait Anger Expression Inventory-2 (STAXI-2)

The STAXI-2 (Spielberger, 1999) provides measures to assess the experience, expression, and control of anger. It comprises 57 items rated on a four-point Likert scale, ranging from 0 (not at all) to 3 (very much indeed). Items are grouped by four scales: the first, State Anger scale, refers to the emotional state characterized by subjective feelings and relies on three more subscales: Angry Feelings, Physical Expression of Anger, and Verbal Expression of Anger. The second scale is the Trait Anger and indicates a disposition to perceive various situations as annoying or frustrating with two subscales—Angry Temperament and Angry Reaction. The third and last scales are Anger Expression and Anger Control. These assess anger toward the environment and oneself according to four relatively independent subscales: Anger Expression-OUT, Anger Expression-IN, Anger Control-OUT, and Anger Control-IN. Alpha coefficients STAXI-2 were above 0.84 for all scales and subscales, except for Trait Anger Reaction, which had an alpha coefficient of 0.76 [Italian version by Spielberger (2004)].

## Statistical Analysis

**Figure 2:**
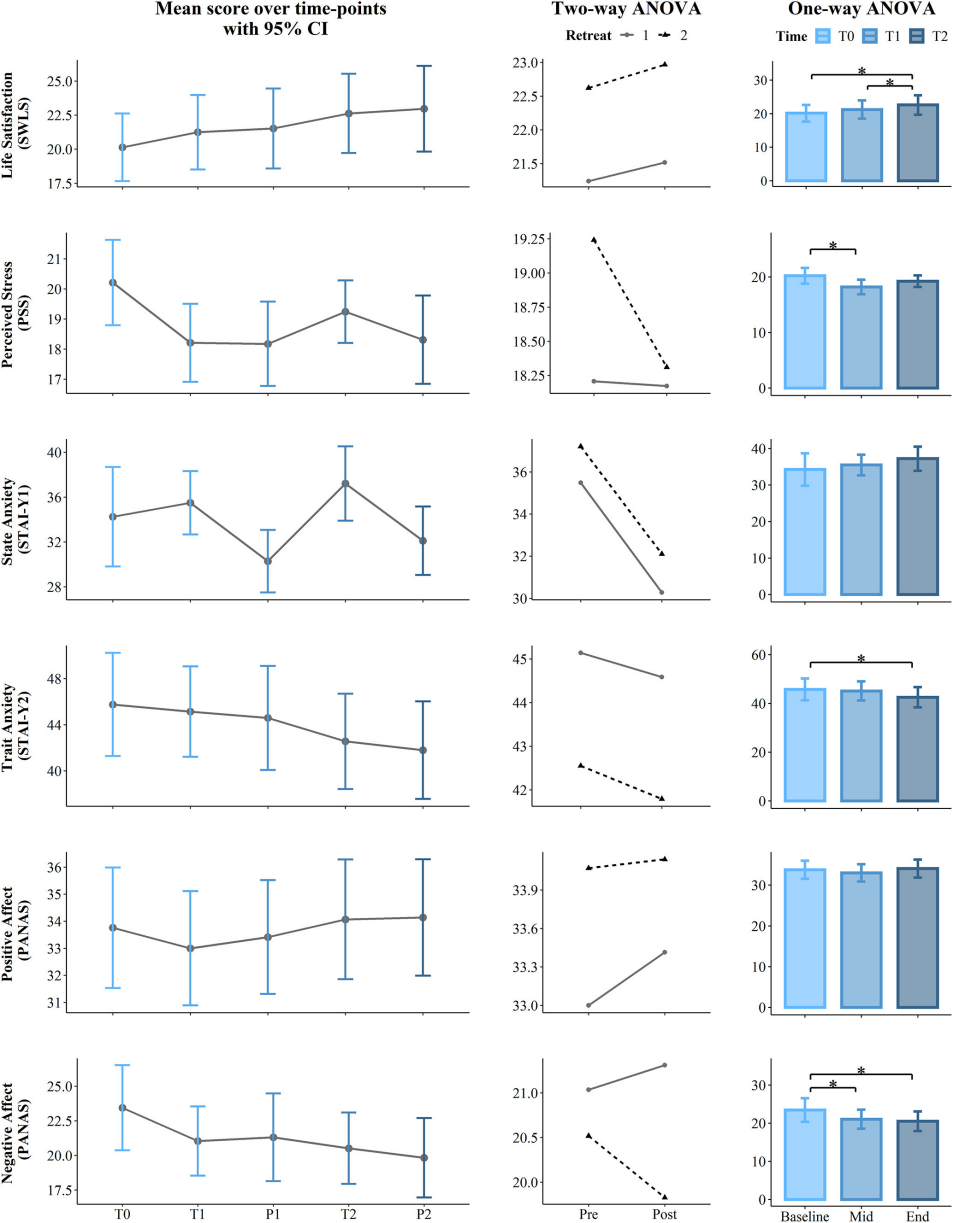
Results of the Satisfaction with Life Scale (SWLS), Perceived Stress Scale (PSS), State and Trait Anxiety Index (STAI), and Positive and Negative Affect Scales (PANAS). The first (left) panel depicts pooled mean raw data per time point estimating 95% confidence interval. The second (central) panel represents changes in pooled mean (*y*-axis) between retreats. The solid line represents retreat 1 and the dotted line denotes retreat 2 derived from the contrasts of the two-way ANOVA. The third (right) panel depicts bar charts representing the changes in mean between the 3 time points derived from the one-way ANOVA. Note that scores are on the *y*-axis and time is on the *x*-axis. Time points legend: baseline (month 1—T0), pre (T1), post (P1), mid-course retreat (month 5—retreat 1), pre (T2), and post (R2) of the final retreat (month 9—retreat 2). Statistical significance, *p < 0.05.

**Figure 3:**
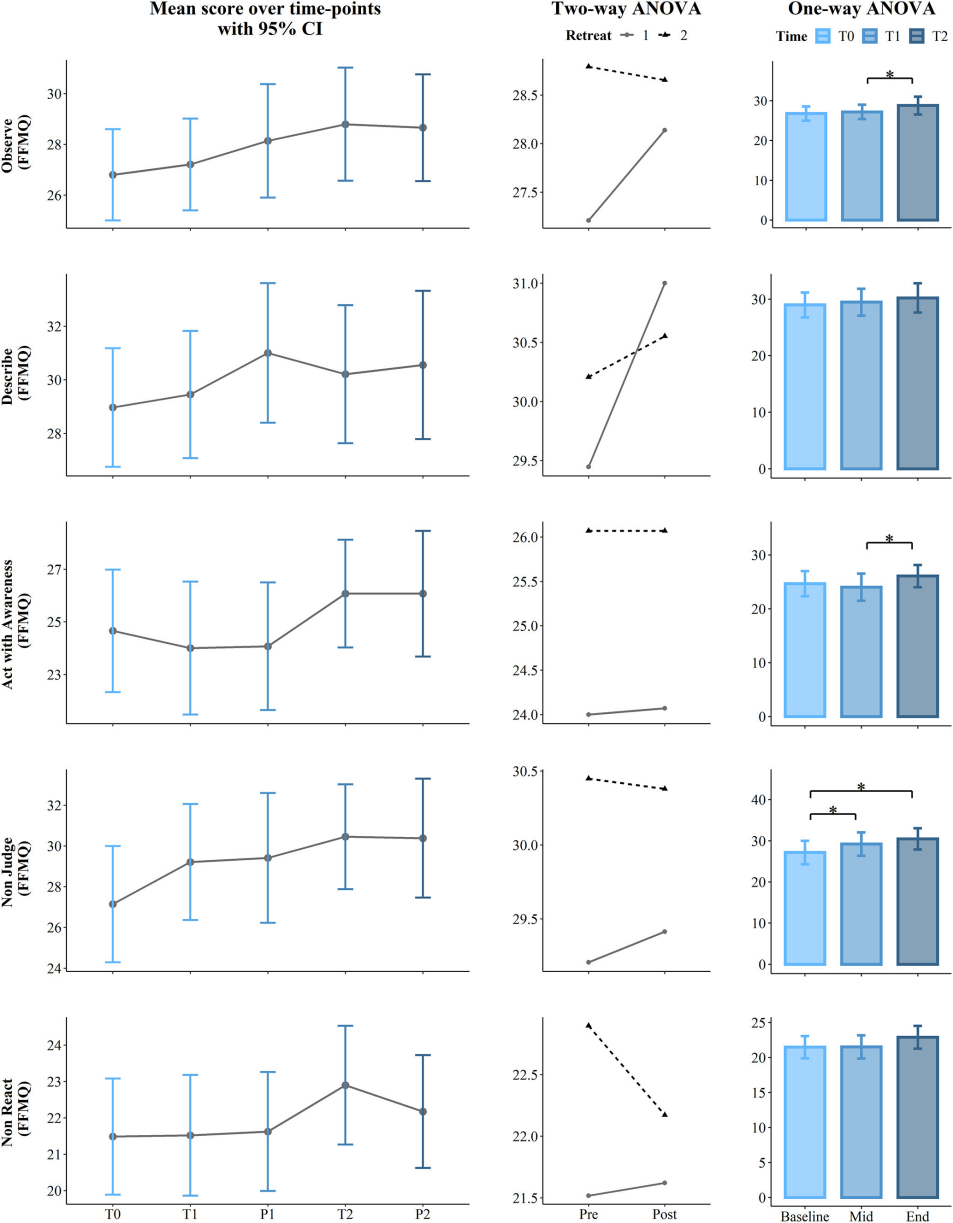
Results for the Five Facet Mindfulness Questionnaire FFMQ (Observe, Describe, Act with Awareness, Non-judge, and Non-react). The first (left) panel depicts pooled mean raw data per time point estimating 95% confidence interval. The second (central) panel represents changes in pooled mean (*y*-axis) between retreats. The solid line represents retreat 1 and the dotted line denotes retreat 2 derived from the contrasts of the two-way ANOVA. The third (right) panel depicts bar charts representing the changes in mean between the 3 time points derived from one-way ANOVA. Note that scores are on the *y*-axis and time id on the *x*-axis. Time points legend: baseline (month 1—T0), pre (T1), post (P1), mid-course retreat (month 5—retreat 1), pre (T2), and post (P2) of the final retreat (month 9—retreat 2). Statistical significance, *p < 0.05.

**Figure 4:**
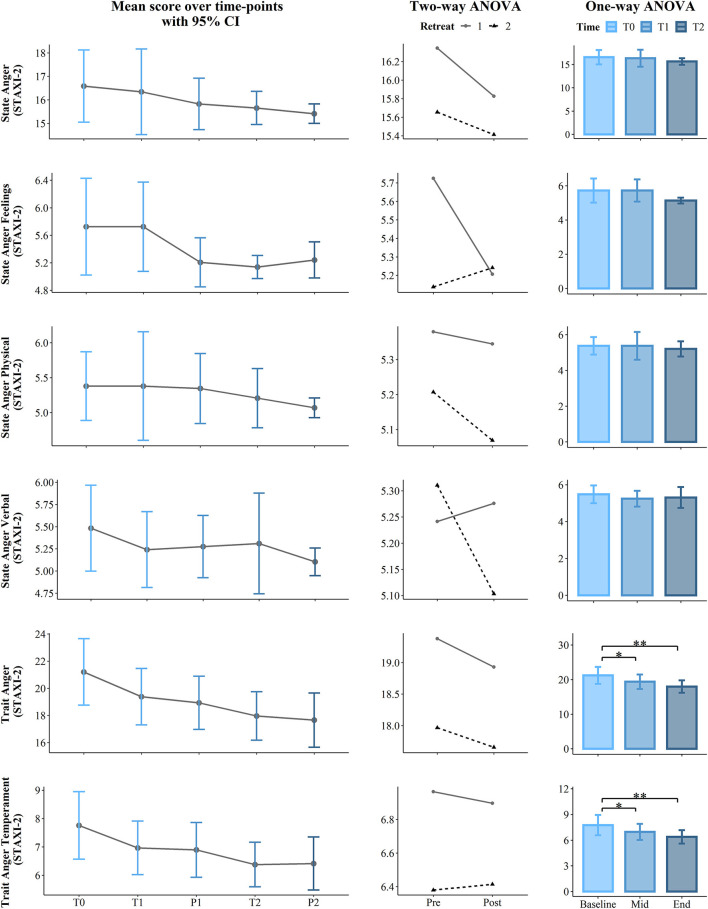
Results of the first part of the State Trait Anger Expression Inventory (STAXI-2): State Anger, State Anger Feelings, State Anger Physical, State Anger Verbal, Trait Anger, and Trait Anger Temperament. The first (left) panel depicts pooled mean raw data per time point estimating 95% confidence interval. The second (central) panel represents changes in pooled mean (*y*-axis) between retreats. The solid line represents retreat 1 and the dotted line denotes retreat 2 derived from the contrasts of the two-way ANOVA. The third (right) panel depicts bar charts representing the changes in mean between the 3 time points derived from one-way ANOVA. Note that scores are on the *y*-axis and time is on the *x*-axis. Time points legend: baseline (month 1—T0), pre (T1), post (P1), mid-course retreat (month 5—retreat 1), pre (T2), and post (R2) of the final retreat (month 9—retreat 2). Statistical significance, **p < 0.01 and *p < 0.05.

**Figure 5:**
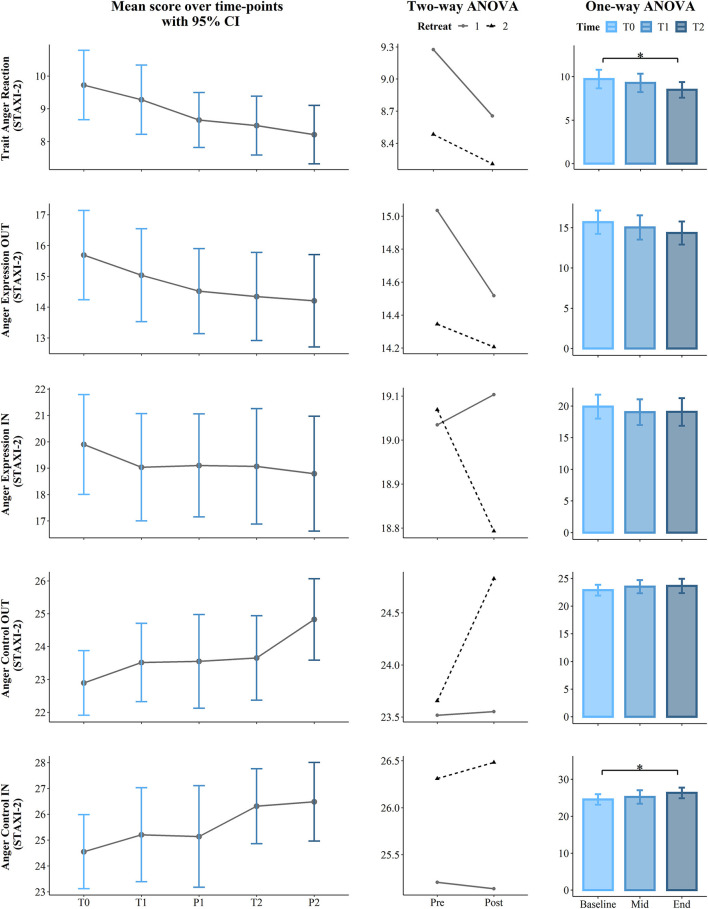
Results from the second part of the State Trait Anger Expression Inventory (STAXI-2): Trait Anger Reaction, Anger Expression-IN, Anger Expression-OUT, Anger Control-IN, and Anger Control OUT. The first (left) panel depicts pooled mean raw data per time point estimating 95% confidence interval. The second (central) panel represents changes in pooled mean (*y*-axis) between retreats. The solid line represents retreat 1 and the dotted line denotes retreat 2 derived from the contrasts of the two-way ANOVA. The third (right) panel depicts bar charts representing the changes in mean between the 3 time points derived from one-way ANOVA. Note that scores are on the *y*-axis and time is on the *x*-axis. Time points legend: baseline (month 1—T0), pre (T1), post (P1), mid-course retreat (month 5—Retreat 1), pre (T2), and post (R2) of the final retreat (month 9—Retreat 2). Statistical significance, *p < 0.05.

The responses on each questionnaire were scored according to their protocols, which resulted in one score per participant and a time point for each of the 22 scale/subscale questionnaires examined. Missing values (<2%) were imputed using the median. Descriptive statistics for all variables were analyzed and are summarized in Table 1 and in the first panel (column) of Figures 2–5. Prior to conducting primary analyses, the distribution of scores on all the dependent variables was evaluated. Because the data were not normally distributed, we used non-parametric tests. Permutation tests are non-parametric tests as they do not rely on assumptions about the distribution of the data and can be used with different types of scales and with a small sample size.

**Table 1:** Descriptive statistics of the depended variables among time points.

			**Retreat 1**				**Retreat 2**			
	**T0 (Baseline)**		**T1 (Pre)**		**P1 (Post)**		**T2 (Pre)**		**P2 (Post)**	
**Variables**	**M**	**SD**	**M**	**SD**	**M**	**SD**	**M**	**SD**	**M**	**SD**
SWLS	20.14	6.52	21.24	7.18	21.52	7.71	22.62	7.63	22.97	8.27
PSS stress	20.21	3.73	18.21	3.41	18.17	3.68	19.24	2.73	18.31	3.86
**STAI**										
Y-1 state	34.24	11.66	35.48	7.42	30.28	7.33	37.21	8.70	32.10	8.03
Y-2 trait	45.76	11.76	45.14	10.33	44.59	11.87	42.55	10.88	41.79	11.14
**PANAS**										
Positive	33.76	5.85	33.00	5.54	33.41	5.53	34.07	5.81	34.14	5.64
Negative	23.45	8.10	21.03	6.56	21.31	8.35	20.51	6.77	19.83	7.56
**FFMQ**										
Observe	26.79	4.73	27.21	4.76	28.14	5.88	28.79	5.86	28.65	5.53
Describe	28.97	5.82	29.45	6.25	31	6.86	30.21	6.78	30.55	7.28
Act aware	24.65	6.12	24	6.64	24.07	6.38	26.07	5.39	26.07	6.28
Non-judge	27.14	7.49	29.21	7.48	29.41	8.37	30.45	6.77	30.38	7.68
Non-react	21.48	4.20	21.52	4.36	21.62	4.30	22.90	4.30	22.17	4.08
**STAXI 2**										
State anger	16.59	4.03	16.34	4.80	15.83	2.88	15.65	1.86	15.41	1.09
S-Ang/F	5.72	1.85	5.72	1.71	5.21	0.94	5.14	0.44	5.24	0.69
S-Ang/P	5.38	1.29	5.38	2.04	5.34	1.32	5.21	1.11	5.07	0.37
S-Ang/V	5.48	1.27	5.24	1.12	5.28	0.92	5.31	1.49	5.10	0.41
Trait anger	21.21	6.43	19.38	5.46	18.93	5.16	17.97	4.70	17.65	5.25
T-Ang/T	7.76	3.12	6.97	2.85	6.90	2.54	6.38	2.06	6.41	2.46
T-Ang/R	9.72	2.79	9.26	2.79	8.65	2.21	8.48	2.37	8.21	2.35
AX-O	15.69	3.81	15.03	3.97	14.51	3.63	14.35	3.76	14.21	3.94
AX-I	19.90	4.97	19.03	5.35	19.10	5.13	19.07	5.75	18.79	5.72
AC-O	22.90	2.58	23.52	3.13	23.55	3.75	23.65	3.37	24.82	3.25
AC-I	24.55	3.77	25.21	4.78	25.14	5.17	26.31	3.82	26.48	4.01

*SWLS, Satisfaction with Life Scale; PSS, Perceived Stress Scale; STAI, State-Trait Anxiety Inventory; PANAS, Positive and Negative Affect Scale; FFMQ, Five Facet Mindfulness Questionnaire; STAXI 2, State Trait Anger Expression Inventory; S-Ang/F, Feeling Angry; S-Ang/V, Feel like Expressing Anger Verbally; S-Ang/P, Feel like Expressing Anger Physically; T-Ang/T, Angry Temperament; T-Ang/R, Angry Reaction; AX-O, Anger Expression-OUT; AX-I, Anger Expression-IN; AC-O, Anger Control-OUT; AC-I, Anger Control-IN*.

The longitudinal effects of the program were analyzed to determine whether scores changed between the start, mid-point (5 months), and the end (9 months) of the course. To achieve this, we compared the main effect of the *program* on the *score*, considering *Time* as a unique factor with three levels: at the baseline (T0), at the pre of the mid-retreat (T1), and at the pre of the final retreat (T2). Here, we used a one-way permutation Repeated Measures Analysis of Variance (RM ANOVA) with the aovperm() function from the Permuco package v. 1.0.2 in R (Frossard and Renaud, 2018), which implements a method from Kherad-Pajouh and Renaud (2014). The difference between the traditional and the permutation ANOVA is that, while the traditional ANOVA tests the equality of the group mean, the permutation version tests the exchangeability of the group observations. In this study, the number of permutations was set to 100,000 and the alpha level was set to 0.05; therefore, the *p*-value was computed as the ratio between the number of permutation tests that have an *F* value higher than the critical *F* value and the number of permutations performed. Effect size estimates were calculated using partial eta squared. *Post hoc* testing used pairwise permutational *t*-tests with the “pairwise.perm.t.test” function from the “RVAideMemoire” package in R (Hervé and Hervé, 2020). To account for Type I errors introduced by multiple pairwise tests and Type II errors introduced by small sample size, we applied the false discovery rate (FDR) correction method of Benjamini and Hochberg (1995) and set statistical significance at *p* = 0.05. Results are summarized in Table 2 and in the third panel (column) of Figures 2–5.

**Table 2:** One-way ANOVA and pairwise comparison results with 100,000 permutations.

	**One-way ANOVA**			**T0–T1**		**T1–T2**		**T0–T2**	
	***F***	***P***	***ηp^2^***	***M diff*.**	***p***	***M diff*.**	***p***	***M diff*.**	***p***
SWLS	5.253	0.008**	0.16	1.1	0.217	1.38	0.032*	2.48	0.016*
PSS stress	5.182	0.009**	0.16	−2	0.02*	1.03	0.163	−0.97	0.163
**STAI**									
Y-1 state	1.381	0.263	0.05	1.24	0.494	1.73	0.491	2.97	0.424
Y-2 trait	5.204	0.009**	0.16	−0.26	0.458	−2.59	0.077	−3.21	0.025*
**PANAS**									
Positive	1.23	0.298	0.04	−0.76	0.487	1.07	0.324	0.31	0.711
Negative	6.222	0.004**	0.19	−2.42	0.021*	– 0.52	0.643	−2.92	0.012*
**FFMQ**									
Observe	4.034	0.023*	0.13	0.42	0.605	1.58	0.038*	2	0.054
Describe	1.389	0.258	0.05	0.48	0.52	0.76	0.493	1.24	0.493
Act aware	3.543	0.036*	0.12	−0.65	0.408	2.07	0.043*	1.42	0.153
Non-judge	6.86	0.002**	0.20	2.07	0.013*	1.24	0.196	3.31	0.013*
Non-react	3.358	0.043*	0.11	0.04	0.997	1.38	0.055	1.42	0.055
**STAXI 2**									
State anger	1.083	0.376	0.04	−0.25	0.783	−0.69	0.783	−0.94	0.456
S-Ang/F	2.289	0.096	0.08	0	1	−0.58	0.115	−0.58	0.238
S-Ang/P	0.415	0.77	0.01	0	1	−0.17	1	−0.17	1
S-Ang/V	0.364	0.733	0.01	−0.24	0.748	−0.07	0.748	−0.17	0.748
Trait anger	8.038	0.001***	0.23	−1.83	0.041*	−1.41	0.07	−3.24	0.002**
T-Ang/T	7.641	0.001***	0.22	−0.79	0.016*	−0.59	0.105	−1.38	0.008**
T-Ang/R	4.482	0.016*	0.14	−0.46	0.287	−0.78	0.131	−1.24	0.023*
AX-O	2.78	0.071	0.09	−0.66	0.325	−0.68	0.234	−1.34	0.118
AX-I	1.129	0.329	0.04	−0.87	0.475	0.04	1	−0.83	0.475
AC-O	1.077	0.349	0.03	−0.62	0.39	0.13	0.87	0.75	0.39
AC-I	3.735	0.03*	0.12	0.66	0.337	1.1	0.171	1.76	0.044*

SWLS, Satisfaction with Life Scale; PSS, Perceived Stress Scale; STAI, State-Trait Anxiety Inventory; PANAS, Positive and Negative Affect Scale; FFMQ, Five Facet Mindfulness Questionnaire; STAXI 2, State Trait Anger Expression Inventory; S-Ang/F, Feeling Angry; S-Ang/V, Feel like Expressing Anger Verbally; S-Ang/P, Feel like Expressing Anger Physically; T-Ang/T, Angry Temperament; T-Ang/R, Angry reaction; AX-O, Anger Expression-OUT; AX-I, Anger Expression-IN; AC-O, Anger Control-OUT; AC-I, Anger Control-IN. Statistical significance: ***p < 0.001, **p < 0.01, *p < 0.05. Effect size follows the Cohen rules of thumb (Cohen, 2013) with 0.01 = small effect, 0.06 = medium effect, and 0.14 = large effect. M. diff = difference in means.

The short-term effects of the contemplative program on each retreat were analyzed to determine whether scores changed post-retreats and whether these changes occurred in both retreats. Thus, we used a two-way permutation RM ANOVA, with the *score* of each scale/subscale as the dependent variable and the within-subject factors *Retreat* (1, 2) and *Condition* (Pre T1/T2, Post P1/P2) as independent variables. Results are summarized in Table 3 and in the second panel (column) of Figures 2–5.

**Table 3:** Results of the two-way permutation RM ANOVAs.

	**Retreat (1–2)**			**Condition (Pre–Post)**			Retreat*Condition		
**Variables**	***F***	***p***	***ηp^2^***	***F***	***p***	***ηp^2^***	***F***	***p***	***ηp^2^***
SWLS	10.701	0.002**	0.16	0.025	0.876	0	0.006	0.937	0
PSS stress	1.539	0.222	0.03	0.386	0.54	0.01	0.9	0.344	0.02
**STAI**									
Y-1 state	2.657	0.109	0.05	8.712	0.004**	0.14	0.002	0.963	0
Y-2 trait	12.526	0.001**	0.19	0.056	0.812	0	0.019	0.893	0
**PANAS**									
Positive	4.244	0.044*	0.07	0.03	0.866	0	0.157	0.693	0.00
Negative	2.423	0.125	0.04	0.014	0.908	0	0.565	0.456	0.01
**FFMQ**									
Observe	7.568	0.008**	0.12	0.077	0.782	0	1.955	0.167	0.035
Describe	0.11	0.74	0	0.31	0.578	0.01	1.664	0.204	0.03
Act aware	15.381	<0.001***	0.22	0	0.987	0	0.004	0.945	0
Non-judge	4.229	0.045*	0.07	0.001	0.97	0	0.066	0.794	0
Non-react	5.799	0.02*	0.10	0.087	0.769	0	1.065	0.308	0.02
**STAXI 2**									
State anger	1.451	0.268	0.03	0.743	0.459	0.03	0.091	0.808	0
S-Ang/F	1.954	0.182	0.03	1.168	0.326	0.04	2.473	0.12	0.04
S-Ang/P	2.918	0.101	0.05	0.251	0.652	0.01	0.155	0.726	0
S-Ang/V	0.075	0.8	0	0.388	0.563	0.01	0.407	0.55	0.01
Trait anger	7.605	0.008**	0.12	1.042	0.354	0.02	0.02	0.888	0
T-Ang/T	5.582	0.022*	0.09	0.066	0.857	0	0.052	0.822	0
T-Ang/R	5.748	0.019*	0.10	0.166	0.708	0	0.443	0.505	0.01
AX-O	1.672	0.199	0.03	0.088	0.77	0	0.241	0.627	0
AX-I	0.183	0.67	0	0.001	0.977	0	0.286	0.595	0.01
AC-O	2.342	0.131	0.04	0.583	0.447	0.01	1.517	0.224	0.03
AC-I	4.955	0.029*	0.08	0.121	0.728	0	0.048	0.829	0

SWLS, Satisfaction with Life Scale; PSS, Perceived Stress Scale; STAI, State-Trait Anxiety Inventory; PANAS, Positive and Negative Affect Scale; FFMQ, Five Facet Mindfulness Questionnaire; STAXI 2, State Trait Anger Expression Inventory; S-Ang/F, Feeling Angry; S-Ang/V, Feel like Expressing Anger Verbally; S-Ang/P, Feel like Expressing Anger Physically; T-Ang/T, Angry Temperament; T-Ang/R, Angry reaction; AX-O, Anger Expression-OUT; AX-I, Anger Expression-IN; AC-O, Anger Control-OUT; AC-I, Anger Control-IN. Statistical significance: ***p < 0.001, **p < 0.01, *p < 0.05. Effect size follows the Cohen rules of thumb (Cohen, 2013) with 0.01 = small effect, 0.06 = medium effect, and 0.14 = large effect.

**Figure 6:**
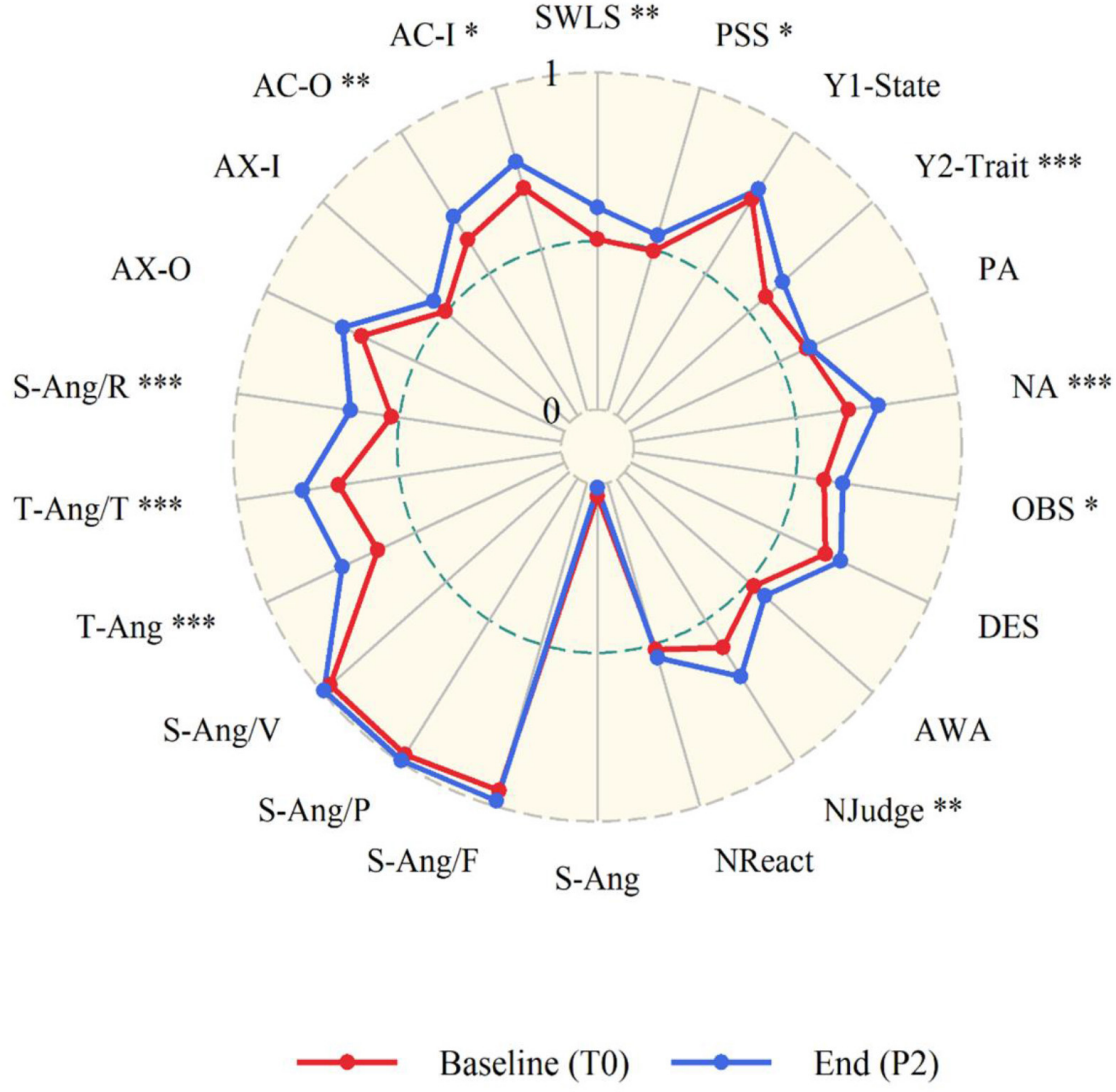
Results of the permutation *t*-test between the start and the end of the course. All values ranged from 0 to 1. Variables: SWLS, Satisfaction with Life Scale; S-Ang/F, Feeling Angry; S-Ang/V, Feel like Expressing Anger Verbally; S-Ang/P, Feel like Expressing Anger Physically; T-Ang/T, Angry Temperament; T-Ang/R, Angry reaction; AX-O, Anger Expression-OUT; AX-I, Anger Expression-IN; AC-O, Anger Control-OUT; AC-I, Anger Control-IN; PSS, Perceived Stress Scale; STAI-Y1, State-Trait Anxiety Inventory—State; STAI-Y2, State-Trait Anxiety Inventory—Trait; PA and NA, Positive and Negative Affect Scales, respectively; OBS, Observe; DES, Describe; AWA, Act with awareness, Njudge, Non-judge; NReact, Non-react. To make consistent that an increase of the specific scale corresponds to an improvement in well-being, negative scales were reversed, namely: PSS, STAI-Y1, STAI-Y2, PANAS-NA, S-Ang, S-Ang/F, S-Ang/P, S-Ang/V, T-Ang, T-Ang/T, S-Ang/R, AX-O, AX-I. Concerning the statistical significance, ***p < 0.001, **p < 0.01, and * *p* < 0.05.

In addition, we explored differences attributed to the course and to the retreats using a paired permutation *t* test with the “perm.t.test()” function in R. We compare those psychological measures at the beginning of the course (T0) with its very end (P2), which coincided with the end of the second retreat. In this way, we illustrate a summary of changes due both to the second retreat and to the whole course. The results are summarized in Table 4 and depicted in a radar plot in Figure 6.

**Table 4:** Overall changes between the start (T0) and the end of the course (P2).

**Variables**	***T***	***M diff*.**	***P***
SWLS	2.797	2.83	0.008**
PSS stress	−2.299	−1.9	0.033*
**STAI**			
Y-1 State	−0.99	−2.14	0.338
Y-2 trait	−4.003	−3.97	<0.001***
**PANAS**			
Positive affect	0.462	0.38	0.687
Negative affect	−3.859	−3.62	0.001***
**FFMQ**			
Observe	2.204	1.86	0.039*
Describe	1.769	1.59	0.095
Act aware	1.752	1.41	0.101
Non-judge	3.02	3.24	0.006**
Non-react	1.182	0.69	0.272
**STAXI 2**			
State anger	−1.756	−1.17	0.111
S-Ang/F	−1.49	−0.48	0.204
S-Ang/P	−1.558	−0.31	0.249
S-Ang/V	−1.692	−0.38	0.187
Trait anger	−4.614	−3.55	<0.001***
T-Ang/T	−4.027	−1.34	<0.001***
T-Ang/R	−3.927	−1.52	<0.001***
AX-O	−1.851	−1.48	0.08
AX-I	−1.437	−1.1	0.175
AC-O	2.861	1.93	0.009**
AC-I	2.555	1.93	0.017*

SWLS, Satisfaction with Life Scale; PSS, Perceived Stress Scale; STAI, State-Trait Anxiety Inventory; PANAS, Positive and Negative Affect Scale; FFMQ, Five Facet Mindfulness Questionnaire; STAXI 2, State Trait Anger Expression Inventory; S-Ang/F, Feeling Angry; S-Ang/V, Feel like Expressing Anger Verbally; S-Ang/P, Feel like Expressing Anger Physically; T-Ang/T, Angry Temperament; T-Ang/R, Angry reaction; AX-O, Anger Expression-OUT; AX-I, Anger Expression-IN; AC-O, Anger Control-OUT; AC-I, Anger Control-IN. Statistical significance: ***p < 0.001, **p < 0.01, *p < 0.05. Effect size follows the Cohen rules of thumb (Cohen, 2013) with 0.01 = small effect, 0.06 = medium effect, and 0.14 = large effect. M. diff = difference in means.

## Results

### Effects of the Program

Results from one-way permutation RM ANOVA showed a statistically significant effect of the program on SWLS at the *p* = 0.008 level over the *Time course* factor with a large effect size (ηp^2^ = 0.16). *Post hoc* analysis revealed that the SWLS score was significantly higher at T2 with respect to T2 (mean difference = 2.48; *p* = 0.016). Similarly, SWLS was higher T2 as compared to T1 (mean difference = 1.38; *p* = 0.032).

Results also provided statistically significant evidence of changes in the PSS over the *Time course* (*p* = 0.009), showing a large effect size (ηp^2^ = 0.16). *Post-hoc* results showed a difference between T0 and T1, revealing that the PSS was significantly lower at T1 (mean difference = −2, *p* = 0.02).

Results revealed a significant effect of the *Time course* for Trait Anxiety (*p* = 0.009, ηp^2^ = 0.16). *Post-hoc* tests revealed a reduction in Trait Anxiety from the start of the course (T0) to the first day of the second retreat (T2) (M diff. = −3.21, *p* = 0.25).

Results also showed a significant effect of the *Time course* for negative affect (*p* = 0.004, ηp^2^ = 0.19). *Post hoc* analysis revealed that contemplative practice led to a reduction in negative affect from the baseline (T0) to the first day of the first retreat (T1) (mean difference = −2.42) and between T0 and first day of the second retreat (T2) (mean difference = −2.92), which differed significantly with *p* = 0.021 and *p* = 0.012, respectively.

Moreover, a significant effect of the *Time course* was found for several subscales of the FFMQ. First, the observe scale was found at the *p* = 0.023 level showing a large effect size (ηp^2^ = 0.13). *Post-hoc* comparisons revealed an increasing capacity to observe one's own thoughts, from the middle of the course (T1) to the first day of the second retreat (T2) (mean difference = 1.58, *p* = 0.038). Likewise, there was a significant difference for the capacity to Act with Awareness (*p* = 0.036, ηp^2^ = 0.12). *Post hoc* comparisons revealed an increased level at T2 as compared to T1 (mean difference = 2.07, *p* = 0.043). The *Time course* had a significant effect on the Non-Judge subscale with a large effect size (*p* = 0.002, ηp^2^ = 0.20). *Post hoc* analysis indicated a significant increase from T0 to T1 (mean difference = 2.07, *p* = 0.013), as well as from T0 to T2 (mean difference = 3.31, *p* = 0.013).

In regard to the STAXI-2, we found *Time course* significant effects on Trait Anger (*p* = 0.001, ηp^2^ = 0.23) and its subscales, Trait Anger Temperament (*p* = 0.001, ηp^2^ = 0.22) and Trait Anger Reaction (*p* = 0.016, ηp^2^ = 0.14). *Post-hoc* comparisons revealed a significance difference on the Trait Anger Scale, which decreased from the beginning of the course (T0) to 5 months later (T1) (mean difference = −1.83, *p* = 0.041) and also from T0 to the end of the course (T2) (mean difference = −3.24, *p* = 0.002). Similarly, State Anger Temperament significantly decreased from T0 to T1 (mean difference = −0.79, *p* = 0.016) and from T0 to T2 (mean difference = −1.38, *p* = 0.008). Additionally, Trait Anger Reaction decreased from T0 to T2 (mean difference = −1.24, *p* = 0.023). Finally, the longitudinal effect of the course on the STAXI-2 led to significant results in the Anger Control-IN subscale over the *Time course* (*p* = 0.03, ηp^2^ = 0.12). Here, *post-hoc* comparisons showed a statistically significant difference between T0 and T2, which increased (mean difference = 1.76, p =.044). For more details, see Table 2 and the third panel (column) of Figures 2–5.

### Effects of the Retreats

Two-way permutation RM ANOVAs showed a significant main effect for *Retreat* on SWLS (*p* = 0.002, ηp^2^ = 0.16), Trait Anxiety (*p* = 0.001, ηp^2^ = 0.19), positive affect (*p* = 0.044, ηp^2^ = 0.07), Observe (*p* = 0.008, ηp^2^ = 0.12), Act with awareness (*p* ≤ 0.001, ηp^2^ = 0.22), Non-Judge (*p* = 0.045, ηp^2^ =.07), Non-React (*p* = 0.02, ηp^2^ = 0.10), Trait Anger (*p* = 0.008, ηp^2^ = 0.12), Trait Anger Temperament (*p* = 0.022, ηp^2^ = 0.09), Trait Anger Reaction (*p* = 0.019, ηp^2^ = 0.10), and Anger Control-IN (*p* = 0.029, ηp^2^ = 0.08). A main effect of the *Condition* (Pre vs. Post) was found only for the State Anxiety scale with *p* = 0.004 and a large effect size (ηp^2^ = 0.14). Analysis results including *F* statistics are summarized in Table 3; a visual representation of the data is presented in the second panel (column) of Figures 2–5.

### Overall Effects of the Course and Retreats

As predicted, permutation *t*-test analysis revealed that participants increased their reported level of SWLS from the start (T0) to the end (P2) of the course (mean difference = 2.83, *p* = 0.008). Two subscales from the FFMQ, namely, the capacity to observe one's own thoughts (mean difference = 1.86, *p* = 0.039) and non-judgmental attitude toward the inner experience (mean difference = 3.24, *p* = 0.006), also significantly increased from the start to the end of the course. On the other hand, the affect linked to the progression from the start (T0) to the very end of the course (P2) was related to a significant decrease in the negative affect (mean difference = −3.62, *p* = 0.001). In the same way, the average level of stress of the sample decreased significantly (mean difference = −1.9, *p* = 0.033) along with a significant decrease of Trait Anxiety (M diff = −3.97, *p* ≤ 0.001). Participants also decreased on almost all STAXI-2 subscales. Here, the results from permutation paired *t*-test reveal a significant difference in scores, which decreased from T0 to P2 on all the subscales of Trait Anger (mean difference = −3.55, *p* ≤ 0.001; Trait Anger Temperament: mean difference = −1.34, *p* ≤ 0.001; Trait Anger Reaction: mean difference = −1.52, *p* ≤ 0.001), with an increased value for the subscales Anger Control-OUT (mean difference = 1.93, *p* ≤ 0.009) and Anger Control-IN (mean difference = 1.93, *p* = 0.017). For more details, see Table 4 and Figure 6.

## Discussion

The aim of this study was to examine the effectiveness of an integrated 9-month mental training program called *The Art of Happiness*, which was developed to increase well-being in a general population. By a range of well-established psychometric assessment tools, we quantified how several psychological well-being variables changed with course attendance. We took into account both the trait effects of the course acting at a long timescale (over the 9-month duration of the full course) and the state effects of intensive retreat experiences acting at a short time scale (over the course of each of the two retreats). Several psychological well-being measures related to states and—more importantly—traits gradually improved as participants progressed from the beginning to the end of the course.

On the one hand, the program produced a significant longitudinal effect (9 months) revealing a progressive increase in the volunteer's levels of life satisfaction and of the capacities to reach non-judgmental mental states, to act with awareness, to non-react to inner experience, and to exercise control over attention to the internal state of anger, in line with other contemplative interventions (Fredrickson et al., 2008; Keng et al., 2011; Baer et al., 2012; Kong et al., 2014). Conversely, after the completion of the program, there were decreases in levels of trait anxiety, trait anger (including both the anger temperament and reaction subscales), and negative affect, showing a progressive reduction during the intervention. These results support prior research that demonstrated the longitudinal positive effects of a multitude of contemplative practices on well-being measures linked to—among others—decreased trait anxiety, trait anger, and negative affect (Fix and Fix, 2013; Khoury et al., 2015; Gotink et al., 2016). Such findings highlight the gradual development of mental states related to subjective well-being in parallel with ongoing contemplative practices over a time scale of months, with a gradual increase of wholesome mental states, and a gradual decrease of unwholesome mental states. Notably, as in other mindfulness interventions (Khoury et al., 2015; Gotink et al., 2016), there was a significant reduction in the level of perceived stress already in the first few months of the program (T0–T1).

Additionally, these results show the specific effects between retreat experiences within the program as an intervention for fostering happiness. Specifically, the retreats had a positive effect on the participants' perceived well-being, which improved between the two retreats (with a 4-month interval). Among other assessed dimensions, between the retreats, there were significantly increased levels of life satisfaction, positive affect, and mindful abilities to act with awareness, to observe, non-react, and non-judge inner experience and the capacity to control anger toward oneself. Conversely, there were significantly lower levels of trait anxiety and trait anger (including both the anger temperament and reaction subscales) between the retreats (over a period of 4 months).

Regarding the very short effects of the course, we highlight significant changes within the first part of the training and prior to the first retreat (T0–T1). Here, some variables related to happiness changed most, suggesting their independence from retreat. Particularly, PSS notably decreased along with negative affect and Trait Anger (the subscale of Angry Temperament), while the capacity of non-judgmental attitude toward the inner experience significantly increased, providing useful information for future interventions.

Moreover, participants' state anxiety significantly decreased in a very short time (5 days), between pre and post of both retreats. These findings are consistent with previous studies, which demonstrated the positive effects of contemplative training and practices on these measures in retreats (Khoury et al., 2017; Howarth et al., 2019; McClintock et al., 2019). In Figure 6, we make a general and integrated comparison between the various psychological measures, comparing the very beginning of the course with its very end, which also coincided with the end of the second retreat. In this way, we illustrate both state changes (due to the second retreat) and trait changes (due to the whole course). This representation allows an integrated view of all the changes that took place at different time scales. This graph might suggest that the only measures that did not change significantly from the beginning to the end of the course are those in which the participants already had a score strongly oriented toward well-being, and therefore with little room for a change. Thus, future studies could take into account individual differences when evaluating happiness programs.

Although the present findings are promising, this study presents several limitations that need to be taken into consideration. The two main limitations rely on the absence of a randomized control group and in the fact that participants were self-selected. This lack of verification makes it difficult to determine whether the results are attributable to the program or to other factors, for example, simply arising due to spending time in a happiness-oriented activity. It is also important to note that despite examining several assessments within persons, the sample size was restricted to 29. Furthermore, responses to the questionnaires may have been biased toward the socially desirable response as the course's staff administered them, and another active group could have controlled for these effects. Consequently, it is recommended to conduct future studies with larger samples and a well-designed and controlled trial, in order to achieve more conclusive findings. Another limitation is that, while all the participants attended the whole course with a comparable (coherent) level of commitment to the practices (including the retreats), we did not verify their course-related activity and practices at home, and therefore, we have no way to check whether they actually did the practice activities at home as suggested during the modules.

Possible new directions of exploration of this study concern the age range of the participants, which, in our case, was limited to middle-aged individuals (39–66), and therefore, the effects on younger or older individuals remain currently unexplored. Another interesting direction would be to conduct follow-up measurements to assess the stability of the longitudinal effects months or years after the end of the program. Finally, while well-being and happiness are individual and subjective narratives of one's life as good and happy (Bauer et al., 2008), and therefore self-assessments through questionnaires are a valid and common tool of investigation, in interventions such as *The Art of Happiness*, it would be appropriate to also explore individual differences, more objective psychophysiological effects, as well as cultural and social aspects influencing the inner model of happiness.

Despite these methodological limitations and still unexplored directions of research, the results described here suggest that *The Art of Happiness* may be a promising program for fostering well-being in individuals, improving mental health and psychological functioning. Longitudinal integrated contemplative programs with retreats offer a unique opportunity for the intensive development of the inner attitudes related to the capacity to be happy, reducing mental health symptoms and improving a more stable eudemonic well-being in healthy adults.

## Data Availability Statement

The data that support the findings of this study are available from the corresponding author, Nicola De Pisapia, upon reasonable request.

## Ethics Statement

The studies involving human participants were reviewed and approved by Ethics Committee of the Sapienza University of Rome. The participants provided their written informed consent to participate in this study.

## Author Contributions

ND, CM, and AR designed the study. ND, CM, LC, and AR collected the data. CR analyzed the data. CR and ND wrote the original draft. All authors edited and reviewed the manuscript.

## Conflict of Interest

The authors declare that the research was conducted in the absence of any commercial or financial relationships that could be construed as a potential conflict of interest.
