# Supplementary material for: The Art of Happiness: An Explorative Study of a Contemplative Program for Subjective Well-Being
Source: Front Psychol. 2021 Feb 11;12:600982. doi: 10.3389/fpsyg.2021.600982 (PMC7904679; doi:10.3389/fpsyg.2021.600982)
Supplement: Supplementary file 1 [file Data_Sheet_1.PDF]

SUPPLEMENTARY INFORMATION

The Art of Happiness: an explorative study of a contemplative program for subjective well-being

Clara Rastelli, Lucia Calabrese, Constance Miller, Antonino Raffone, Nicola De Pisapia

The Art of Happiness: General program of activities

The Art of Happiness was an intensive nine-month contemplative program for voluntary participants with little or no experience in meditation. The program consisted of seven modules (the first module was five days in length, the others two days in length, usually over weekends from Friday evening through Sunday) and two retreats (an intermediate retreat lasting six days and a final retreat lasting five days). Modules and retreats took place roughly on a monthly basis and in the following order: modules 1 to 4, followed by the intermediate retreat, then modules 5 to 7, followed by the final retreat. In this way, the entire program lasted approximately nine months.

The general types of activities of each module were as follows:

Presentations of core course subject matter: Lectures with slides on the specific topics of each individual module presented by a Buddhist teacher from the Tibetan tradition. During these presentations, participants could intervene and ask questions for clarification. The length of each presentation could vary between 30 and 60 minutes depending on the specific topic and the questions.

Scientific presentations: Lectures with slides presented by a cognitive neuroscientist. During these presentations, participants could intervene and ask questions for clarification. The length of each scientific presentation could vary between 50 and 75 minutes depending on the specific topic and the questions.

Contemplative practices: Various types of contemplative activities, ranging from individual reflections, a contemplative art activity, guided meditations on relevant topics, basic focused and awareness meditations of various types. All meditations were derived from the Buddhist contemplative traditions, both Mahayana and Theravadin.

Various individual or group activities, including:

- *Mindful Dialogue in pairs*: Participants were divided randomly into pairs. Each person shared with their partner for a total of about five minutes each on a specific topic or question indicated by the teacher using the skills of mindful speaking and mindful listening. The structure of the discussion was not a conversation *per se*. The "speaking" participant was asked to speak thoughtfully, attentively and with deliberation; the "listening" participant was asked to listen mindfully, without interrupting or intervening, without judgment, with total openness and acceptance.
- *Small group or Plenary group sharing/discussions:* Everyone was free to ask questions of the instructors or to share thoughts or experiences. Small group sharing activities focused on a specific question or questions. Plenary group discussions were often more open.

Practice activities at home: The teachers gave a series of indications on activities or exercises that the participants could carry out at home between one module and another.

Course materials

The main bibliographic source accompanying the program was the book *The Art of Happiness* by the Dalai Lama and Howard Cutler. This book served as the content basis for the course. As such, the inner structure of the course, the subject matter covered, the order of topics presented, and so forth, followed the structure of this root text (see the following outline of the course content for the relevant readings from this book associated with each individual module).

To supplement the main presentations of the core course subject matter, relevant TED (and other) videos of talks by a variety of experts in the fields of science, psychology, contemplative practices, sociology and other disciplines were regularly used to expand the discussion topics and offered a variety of points of view on the subject of focus. In addition, numerous citations, extracts, and poetry from both Western and Eastern philosophical and spiritual traditions were shared during the sessions. All slides and audio-recordings of all the lectures, as well as the audio-recordings of the guided meditations, were made available to the participants at the end of each module.

Intermediate and final retreats

The intermediate and final retreats of this course served as opportunities for participants to deepen their understanding and inner experience of the course content. During each retreat, the preceding modules had a full day dedicated to that module's subject matter. In other words, the intermediate retreat occurred after the completion of modules 1 through 4, and thus, the first full day of retreat was dedicated to module 1, the second day to module 2, and so forth. The final retreat occurred after the completion of modules 5 through 7 and was organized similarly.

Each day dedicated to a module was then organized in the following way:

- Mornings: Practice of total silence from time of awakening until after lunch. Beginning the day with motivation meditation, followed by a series of guided meditations on topics and contemplative methods relevant to the day's module, exploring more deeply the subject matter and its application to daily life.
- Afternoons: Beginning with a specific topic of individual reflection pertaining to the application of the subject matter to a particular aspect of life, followed by small-group work on that topic, sharing and diving deeper into one's personal experience through discussion with 2-4 other course participants. The small-group work was followed by a plenary session in which each small group presents any insights or particularly impactful contents that arose in their group. The afternoon then ended with a final concluding meditation to allow the day's understandings to settle and stabilize in the minds of the participants.

In addition to the above, during the final retreat, participants were also divided into small groups in order to create a final presentation to the whole group of their experience of the course in whatever form they choose: theatre, songs, small films, poetry or other creative formats.

Here is a detailed list of all the specific activities and topics for each module and retreat.

<u>MODULE 1</u>

Topic: The purpose of life and authentic happiness (*The Art of Happiness,* chaps. 1–4)

- LECTURES AND PRESENTATIONS
  - **Presentations of core course subject matter:**
    1. The purpose of life and authentic happiness. Definitions of happiness; why happiness matters; the benefits of happiness; what happiness is not; differences between happiness and pleasure; happiness and selfishness; the habit of happiness.
    2. The sources of happiness. Where does our happiness come from.
    3. The positive states of mind: joy, patience, enthusiasm, gratitude, compassion, openness, hope, kindness, tenderness, equanimity, love, generosity, honesty, courage.
    4. Meditation as a tool for training the mind, as a process of familiarization with the inner causes of well-being. Primary components of meditation: steady attention and mindfulness. Types of meditation: focused meditation and analytical meditation. Constructive meditations and deconstructive meditations.
    5. Introduction to the power of awareness of our motivations. The role of motivation in life and in well-being and unhappiness. The power of cultivating healthy motivations in life.
    6. The relationship between ethical attitudes and actions and personal well-being. Ethics as an inner attitude upon which outer actions depend.
  - **Scientific presentations:**
    1. Mental capital and well-being. The human nervous system. Anatomy of the central and peripheral system. Neuroscience and psychology. Brain plasticity.
    2. The psychology and neuroscience of happiness. The mechanisms of pleasure and the brain circuits of reward. The neurotransmitters of happiness: dopamine, serotonin, oxytocin and endorphins. Positive psychology: basic ideas and limits. Effects of positive and negative emotions on cognition. Flow as an optimal experience. Can happiness be learned?
    3. Neuroscience and meditation. Mind wandering: negative and positive aspects. The brain networks of mental wandering. The default-mode network. Definition of mindfulness. The practice of mindfulness in the Western world. A neurocognitive model of the practice of

breathing meditation. Psychophysiological effects of focused meditation. Effects of mindfulness on the brain and behavior. What is introspection. Analytical Meditation and Compassion Meditation.

- CONTEMPLATIVE PRACTICES
    - **Individual reflections**
        1. Our starting point: What exactly do I think happiness is?
        2. Initial exploration of motivation: Why am I here? What brought me to participate in this course? What would I like to receive from this course?
        3. What is my happiness made up of? What conditions, what factors, what elements? (preliminary contemplation before creating a personal mind map of "My Happiness".)
        4. Three questions within the context of maximizing one's own well-being: (1) What aspects of myself and my life bring me real satisfaction and well-being? (2) What qualities or characteristics of myself that contribute to my happiness would I like to strengthen/reinforce? (3) What characteristics of myself that diminish my well-being would I like to transform or eliminate?
    - **Basic Meditations** *(3-4 short sessions distributed during each day of the course)*
        1. Sustained mindful attention on various objects: the breath, bodily sensations, thoughts, sounds
        2. Centering and grounding meditations
- INDIVIDUAL OR GROUP ACTIVITIES
    - **Mindful dialogue/mindful listening (pairs)**
        1. What is true happiness?
        2. Where does my happiness come from?
    - **Plenary group discussions**
        1. What is true happiness? (Sharing from the Mindful dialogue activity)
        2. Open Q+A sessions (3)
    - **Contemplative art activity**
        1. Drawing a mind map of "My Happiness".
- PRACTICE ACTIVITIES AT HOME
    - **Daily practices:**
        1. At the end of each day, find three positive things that happened (that one did or experienced, or that others did) during the day, contemplating the contributing circumstances, causes and conditions. Generating appreciation and gratitude.
        2. Cultivating a positive motivation at the start of each day through inner reflection.
        3. Daily diary of personal reflections.

MODULE 2

Topic: Empathy and compassion (*The Art of Happiness,* chaps. 5–7)

- LECTURES AND PRESENTATIONS
    - **Presentations of core course subject matter:**
        1. Defining empathy and kindness. Defining compassion as an inner attitude. The differences between empathy and compassion. The components of compassion. The benefits of compassion. Emerging compassion and active compassion. Differentiating genuine compassion and false compassion. What is and is not compassion.
        2. The obstacles to compassion: indifference, distraction, pride, hatred, selfishness, discrimination.
        3. The development and practice of compassion: inner methods (meditations) and behavioral methods (casual daily acts of kindness, reflections). The triad of compassion + wisdom + ability/skillful means. Compassion in action, compassion rooted in wisdom; the ability to help or not; maintaining a compassionate attitude even when we are impotent and cannot help.
    - **Scientific presentations:**
        1. Empathy in psychology and neuroscience. When empathy is born in the history of life. Empathy and parental attachment. The role of the recognition of emotions. Basic emotions. The role of the amygdala. Facial expressions and the role of the muscles of the eye

orbits. The theory of simulation. Sociopathy. Contagion of mental states. Taking the cognitive perspective of others. The theory of mind. Autism.
2. Neuroscience and compassion. Differences between emotional empathy and compassion. Brain plasticity and compassion. Psycho-neuro-endocrinological effects of compassion.

- CONTEMPLATIVE PRACTICES
  - **Individual reflections**
    1. Continuing exploration of motivation: Observing actual motivations at work. Transforming present motivations by applying broader, more altruistic perspectives.
    2. Cultivating positive motivation at the start of the day.
    3. Individual reflection on moments of genuine compassion or false compassion in one's own life experiences.
    4. Reflecting on moments in life when there was someone who needed our help. How did we help? Did we use wisdom? Compassion? Skill?
  - **Basic Meditations** *(2-3 short sessions distributed during each day of the course preparatory to guided meditations or other activities)*
    1. Sustained mindful attention on various objects: the breath, bodily sensations, thoughts, sounds
    2. Centering and grounding meditations
  - **Guided Meditations**
    1. Cultivating compassion
    2. The kindness of others
- INDIVIDUAL OR GROUP ACTIVITIES
  - **Small group sharing**
    1. Our starting point: What is compassion, why do we need compassion, what the world would be like without compassion, what our individual life would be like without compassion.
    2. Where does my happiness come from?
  - **Plenary group discussions**
    1. What is true happiness? (Sharing from the Mindful dialogue activity)
    2. Open Q+A sessions (2)
  - **Mindful dialogue/mindful listening (pairs)**
    1. "I find it hard to be compassionate when ...."
- PRACTICE ACTIVITIES AT HOME
  - **Daily practices:**
    1. Consciously do at least one gratuitous act of kindness each day.
    2. Cultivating a positive motivation at the start of each day through inner reflection.
    3. Cultivate a short daily meditation practice (self-awareness, self-care, grounding, centering, sustained attention)
    4. If desired, continue the daily practice of Three Positive Things.
    5. Daily diary of personal reflections.

<u>MODULE 3</u>

Topic: Transforming life's suffering (*The Art of Happiness,* chaps. 8–11)

- LECTURES AND PRESENTATIONS
  - **Presentations of core course subject matter:**
    1. Recognizing suffering. Manifestations of suffering: birth, old age, illness, death, separation, uncertainty, dissatisfaction.
    2. Changing perspective on suffering. Courage, openness, equanimity, knowledge, intelligence, wisdom, compassion.
    3. Methods for how to change perspective. Benefits of changing how we relate with our problems.
  - **Scientific presentations:**
    1. When does suffering start in a human being? The function of crying in babies. Physical suffering and psychological suffering. Social exclusion. The importance of pain. Nociceptive system. Congenital insensitivity to pain. Addiction. Positive and negative reinforcements. Association learning. Dopamine and rewards. Hedonic dysregulation and mechanisms of

pleasure. The placebo effect: experimental evidence and fields of application in the control of pain.
2. Emotional regulation. How to transform emotions. Intervention strategies in emotional regulation. The distancing and the change of perspective, Meditation and pain. Meditative practices for addictions.

- CONTEMPLATIVE PRACTICES
  - **Individual reflections**
    1. Continuing exploration of motivation: Observing actual motivations at work. Transforming present motivations by applying broader, more altruistic perspectives.
    2. Cultivating a positive motivation for the day.
    3. Contemplating the presence of uncertainty and dissatisfaction in life.
    4. Reflecting on a specific problem in life with different eyes. Practicing changing perspective.
  - **Basic Meditations** *(2-3 short sessions distributed during each day of the course preparatory to guided meditations or other activities)*
    1. Sustained mindful attention on various objects: the breath, bodily sensations, thoughts, sounds
    2. Centering and grounding meditations
  - **Guided Meditations**
    1. Forgiving ourselves.
    2. Taking on the suffering of self and others and giving relief (Tong-len)
- INDIVIDUAL OR GROUP ACTIVITIES
  - **Plenary group discussions**
    1. Open Q+A sessions (3)
  - **Mindful dialogue/mindful listening (pairs)**
    1. Our starting point: How do you react when you have problems in your life?
- PRACTICE ACTIVITIES AT HOME
  - **Daily practices:**
    1. Cultivating a positive motivation at the start of each day through inner reflection. Consciously cultivate each day a gentle, open, friendly and kind attitude toward oneself and others.
    2. Cultivate a short daily meditation practice (self-awareness, self-care, grounding, centering, sustained attention)
    3. If desired, continue the daily practice of Three Positive Things; and/or the practice of One Kind Action.
    4. Daily diary of personal reflections.

<u>MODULE 4</u>

Topic: Working with the disturbing emotions: Anger and hatred (*The Art of Happiness*, chaps. 12–13)

- LECTURES AND PRESENTATIONS
  - **Presentations of core course subject matter:**
    1. The disturbing emotions. The three poisons: ignorance, aversion and attachment. The mental afflictions associated with aversion. What arises from aversion: impatience, irritation, frustration, disdain, fury, resentment, intolerance, malevolence, resentment, envy. The mechanisms of reactivity. Anger, definition and its effects. Effects of anger on decisions.
    2. Can mental afflictions be eliminated? Reasons to be able to say yes. Antidotes to anger: awareness, patience, courage, change of perspective, love. The origin of patience and love. Inner states following the practice of mental training.
  - **Scientific presentations:**
    1. Anger and the brain. Psychological definition of anger. Causes of anger. Amygdala and anger. The stress reaction. Sympathetic and parasympathetic nervous system. Health consequences of anger attacks. Hunger and anger. Serotonin. Psychological pathologies associated with anger.
    2. Intervention on anger. The neurocognitive model of anger and points of intervention. Management of anger and neuroplasticity. Breaking the automatic responses to anger. The power of distraction. Angry rumination. Social outbursts of anger. Exercise and anger. The power of reframing and the change of perspective. Prefrontal cortex, imagination, serotonin in relation to patience.

- CONTEMPLATIVE PRACTICES
  - **Individual reflections**
    1. Deepening contemplation of motivation and cultivating wholesome motivations in life; at the start of each day.
    2. Exploring our inner reactivity; reflecting on specific experiences of anger, deconstructing moments of anger.
    3. Deconstructing a moment when another person has made us angry.
  - **Basic Meditations (As in previous modules:** *2-3 short sessions distributed during each day of the course preparatory to guided meditations or other activities)*
  - **Guided Meditations**
    1. Exploring the angry mind.
    2. The harmful effects of anger.
    3. Is it possible to reduce or even eliminate the anger in my mind? Is it worthwhile to do so? Why or why not?
    4. Forgiving oneself, forgiving the other.
- INDIVIDUAL OR GROUP ACTIVITIES
  - **Small group sharing**
    1. Our starting point: How do anger and hatred manifest in your life? What effect do they have? Do you think this is a problem, or not?
  - **Plenary group discussions**
    1. Open Q+A sessions (3)
  - **Mindful dialogue/mindful listening (pairs)**
    1. "The harmful results of anger that I have directly experienced are..."
- PRACTICE ACTIVITIES AT HOME
  - **Daily practices:**
    1. Consciously cultivate each day a gentle, open, friendly and kind attitude toward oneself and others.
    2. Cultivate a positive motivation at the start of each day through inner reflection.
    3. Cultivate a short daily meditation practice (self-awareness, self-care, grounding, centering, sustained attention)
    4. If desired, continue the daily practice of Three Positive Things; and/or the practice of One Kind Action.
    5. Daily diary of personal reflections.

<u>INTERMEDIATE RETREAT</u>

Day 1: Module 1 – The purpose of life and authentic happiness

- MEDITATIONS
  1. Reflecting on motivation; cultivating wholesome motivation for the day
  2. Reflecting on the nature and importance of genuine happiness in life.
  3. Reflecting on the difference between pleasure (hedonistic happiness) and happiness (eudaimonia) and the significance of these distinctions.
  4. Reflection: All living beings wish for happiness and wish to avoid suffering. Is this true? What are the deeper consequences of this statement?
- AFTERNOON TOPIC FOR REFLECTION AND DISCUSSION
  1. Bring to mind specific personal experiences of both hedonistic pleasure and eudemonistic happiness, of the benefits or damage created on such occasions to both self and others. Comparing these experiences and clarifying their distinctions. Exploring the ramifications of these differences to one's own personal growth as a human being, one's own well-being, etc.

Day 2: Module 2 – Empathy and compassion

- MEDITATIONS
  1. Focusing the mind; centering and grounding; focusing on the breath.
  2. Reflecting on motivation; cultivating wholesome motivation for the day
  3. Equanimity
  4. Loving kindness
- AFTERNOON TOPIC FOR REFLECTION AND DISCUSSION

1. Reflecting on the differences between empathy and compassion. Bringing to mind specific personal experiences of empathy without compassion, compared with experiences of the presence of compassion. Feeling the differences in these experiences. Analyzing and exploring those differences and the various causes and conditions giving rise to either empathy, or compassion, or both. Reflecting on one's behavior and reactions in each instance.

Day 3: Module 3 – Transforming life's suffering

- MEDITATIONS
  1. Focusing the mind; centering and grounding; focusing on the breath.
  2. Reflecting on motivation; cultivating wholesome motivation for the day
  3. Opening to the uncomfortable and painful with Yes
  4. The practice of self-compassion, opening to one's own pain and discomfort with kindness
  5. Taking on the suffering of self and others and giving relief (Tong-len)
- AFTERNOON TOPICS FOR REFLECTION AND DISCUSSION (first in pairs, then in triads)
  1. (Pairs) Reflecting on and sharing with a partner one or more personal painful experiences and occasions in which one was able or not able to reframe the moment, change perspective and transform the experience into something more useful and less harmful. Reflecting on what one can learn/gain from the painful and difficult experiences in life. What inner abilities or qualities do we develop thanks to the difficulties we experience in life and overcome?
  2. (Triads) Bring to mind a personal experience of being able to meet with fortitude and balance a moment of pain or suffering in life, transforming the experience into an important and worthwhile challenge in your life's path. Reflect on the experience; deconstruct and analyze what happened, what were the inner and outer causes and conditions that contributed to the experience evolving in that way. What did you learn? What did you feel?

Day 4: Module 4 – Working with the disturbing emotions: Anger and hatred

- MEDITATIONS
  1. Focusing the mind; centering and grounding; focusing on the breath.
  2. Reflecting on motivation; cultivating wholesome motivation for the day
  3. Reframing a specific situation to alleviate anger
  4. Forgiveness
  5. Taking on the suffering of anger and hatred of self and others and giving relief (Tong-len)
- INDIVIDUAL CONTEMPLATIVE ACTIVITY
  1. Starting with a calm reflective meditation on a specific situation/person toward which one is experiencing anger. Then, writing a letter to that person, the object of one's anger, explaining one's own feelings, experiences, and how one would prefer to feel and what kind of situation would be preferable, taking responsibility for one's own feelings and perceptions, being as totally honest about one's own personal view of the situation.
     Again, a calming meditation, finding one's own inner state of balance and letting go of the above emotions. Then, writing a letter to oneself as if one is the other person, putting oneself in the shoes of the other person and responding to the first letter written above, responding as honestly and openly as possible, seeking to feel from the side of the other person as much as possible.
     A final centering meditation, letting go of any emotions generated. In the resulting inner space, reflect upon what one has learned or experienced in this exercise and what lessons one wishes to keep close in the heart and apply in life.
- AFTERNOON TOPIC FOR REFLECTION AND DISCUSSION
  1. Bringing to mind a situation in which one was able to NOT get angry with someone in a conflictual situation but instead was able to maintain inner calm, take a broader perspective, place oneself in the position of the other and not allow anger to take over. Deconstruct and analyze that situation. What made it possible? What causes and conditions contributed? How did the situation conclude? What important or meaningful things did one learn from the experience that one wishes to hold onto and apply in life?

<u>MODULE 5</u>

Topic: Working with the disturbing emotions: The image of self (*The Art of Happiness,* chap. 14)

- LECTURES AND PRESENTATIONS
  - **Presentations of core course subject matter:**

1. The different images that we hold of ourselves: the person we are when we are all alone, the person that we present to the world. The concept of "I" as a reified thought in the mind that has limited reality. The "person" as an ever-changing flow, not fixed, always in flux. The suffering of clinging to a reified image of oneself and ignoring/remaining ignorant of one's constant changing "self".
2. Ignorance, one of the 3 basic causes of suffering, as rooted in this basic mistaken view of self. The natural but limited view of being at the center of the world. Self-centeredness as inhibiting a recognition of our fundamental interconnectedness, interdependence, and therefore as a basis for ongoing suffering.
3. The conclusions and interpretations that we reach based on that reified image of self and their consequences: over-estimation and under-estimation; self-criticism and self-hatred. The inner critic; our inner monologue regarding ourselves and the world. Creating a false world through description and interpretation; how this brings suffering and difficulties. Antidotes to these false views through contemplating impermanence and change; equanimity and acceptance; forgiveness; self-care and loving kindness; letting go; reframing; "It's not all about me".

- **Scientific presentations:**
  1. Mythological origin of individual identity. When is the "I" born in the history of life and in individuals. Self-awareness. Difference between the concepts of (self)-awareness, selfhood, me and person. Cognitive psychology of the self.
  2. Properties of the self-image: perspective, unity, coherence, independence, identity. Construction of these properties and their deconstruction. Is the self an illusion for neuroscience? Person and hypocrisy. Self-promotion. Autonomy of the self. The brain networks of the self. Hypo-egoic phenomena. The transcendence of oneself.

- CONTEMPLATIVE PRACTICES
  - **Individual reflections**
    1. Deepening contemplation of motivation and cultivating wholesome motivations in life; at the start of each day.
    2. Our starting point: Individually reflect on three points: Describe the person (yourself) you present to the world. Describe the person (yourself) that you do not present to the world. Describe the differences between these two.
    3. Reflecting on what aspects of yourself are you afraid to reveal to others. What aspects of yourself are you afraid to recognize and embrace. What are your fears and vulnerabilities.
  - **Basic Meditations (As in previous modules:** *2-3 short sessions distributed during each day of the course preparatory to guided meditations or other activities)*
  - **Guided Meditations**
    1. Forgiving oneself.
    2. Self-compassion.
- INDIVIDUAL OR GROUP ACTIVITIES
  - **Plenary group discussions**
    1. Open Q+A sessions (3)
- PRACTICE ACTIVITIES AT HOME
  - **Daily practices:** *As listed in Module 4. No new at-home practices for this module.*

<u>MODULE 6</u>

Topic: Life and death (*The Art of Happiness*, no specific corresponding chapter)

- LECTURES AND PRESENTATIONS
  - **Presentations of core course subject matter:**
    1. The value of reflecting on and cultivating a constant awareness of death. Awareness of death as a friend in cultivating our best human qualities, in giving the deepest meaning to our human lives. Overcoming the fear of death through knowledge and understanding.
    2. The certainty of death. The story of Kisagotami. How the sense of death can help to appreciate the value of life. The importance of inner resources to face death. The moment of death can come at any time. There is no guaranteed lifespan; we can die at any time. Every moment as a moment of death and a moment of rebirth. The pervasiveness of impermanence: all things change; all things die. Finding beauty in impermanence.

- **Scientific presentations:**
  1. Consciousness from the scientific point of view. A reductionist approach to consciousness. Easy and hard problems of consciousness. What is subjectivity? Towards a scientific theory of consciousness. Theories based on information processing. Has science become panpsychist? Elements of Integrated Information Theory. Consciousness in waking, in sleep and in death. Studies of Near Death Experience, medical and psychological research.

- CONTEMPLATIVE PRACTICES
  - **Individual reflections**
    1. Deepening contemplation of motivation and cultivating wholesome motivations in life; at the start of each day.
    2. Our starting point: Do you think about your death from time to time? What kind of thoughts do you have? How do you view death? How do you feel when you think about death?
    3. Reflect on the hour just passed as if it were the last hour of your life. You realize that you are dead and ask yourself: Are you satisfied with how you lived the last hour of your life?
    4. Reflection: When you reach the moment of death and look back on your life, what kind of life do you want to have lived? What kind of person do you want to be when you reach the end of this life? Every action now creates that person or takes us away from that person. Remembering that we will die and that we are constantly creating ourselves helps us to be more self-aware and to make wiser, more thoughtful decisions.
  - **Basic Meditations (As in previous modules:** *2-3 short sessions distributed during each day of the course preparatory to guided meditations or other activities)*
  - **Guided Meditations**
    1. Imagining experiencing the death process (according to the Tibetan Buddhist tradition).
- INDIVIDUAL OR GROUP ACTIVITIES
  - **Plenary group discussions**
    1. Open Q+A sessions (3)
- PRACTICE ACTIVITIES AT HOME
  - **Daily practices:** *As listed in Module 4. No new at-home practices for this module.*

<u>Module 7</u>

Topic: Cultivating the spiritual dimension of life: A meaningful life (*The Art of Happiness,* chap. 15)

- LECTURES AND PRESENTATIONS
  - **Presentations of core course subject matter:**
    1. The importance of understanding the potential of human beings. Recognition of the importance of inner change. What we need to live a meaningful life. Ethics, concentration and wisdom. The six perfections: morality, generosity, patience, joyful effort, concentration, and wisdom. The archetype of the hero. Can we too be heroic in order to find meaning in life?
  - **Scientific presentations:**
    1. The scientific study of wisdom. Intelligence, creativity and wisdom in psychological research. Wisdom in Western Philosophy. The four cardinal virtues and the three theological virtues in the history of Western thought. The Berlin model of wisdom. How to measure wisdom. Ideas for a brain model of wisdom. Wisdom and age.
- CONTEMPLATIVE PRACTICES
  - **Individual reflections**
    1. Deepening contemplation of motivation and cultivating wholesome motivations in life; at the start of each day.
    2. Our starting point: What is a meaningful life for you? Is a meaningful life the same as a spiritual life?
  - **Basic Meditations (As in previous modules:** *2-3 short sessions distributed during each day of the course preparatory to guided meditations or other activities)*
  - **Guided Meditations**
    1. Meditation on the potential and value of this precious human life.
    2. How to cultivate joy.
- INDIVIDUAL OR GROUP ACTIVITIES
  - **Small group sharing**

        1. Our starting point: What is a meaningful life for you? Is a meaningful life the same as a spiritual life?
    - **Plenary group discussions (more open discussion time in this last module)**
        1. Open Q+A sessions (4)
- PRACTICE ACTIVITIES AT HOME
    - **Daily practices:** *As listed in Module 4. No new at-home practices for this module.*

<u>FINAL RETREAT</u>

Day 1: Module 5 – Working with the disturbing emotions: The image of self

- MEDITATIONS
    1. Focusing the mind; centering and grounding; focusing on the breath.
    2. Reflecting on motivation; cultivating wholesome motivation for the day
    3. The impermanence of oneself
    4. What does it mean to ***truly*** take care of oneself?
    5. Self-compassion
- AFTERNOON TOPIC FOR REFLECTION AND DISCUSSION
    1. How can we truly see ourselves moment by moment, accept ourselves moment by moment, and take care of ourselves moment by moment throughout life?

Day 2: Module 6 – Life and death

- MEDITATIONS
    1. Focusing the mind; centering and grounding; focusing on the breath.
    2. Reflecting on motivation; cultivating wholesome motivation for the day
    3. The impermanence of oneself: Dying in every moment
    4. The uncertainty of when death will claim us: The freedom of a constant awareness of death
    5. Self-compassion
- AFTERNOON TOPIC FOR REFLECTION AND DISCUSSION
    1. You have one month to live. What now has the most importance? What do you wish to protect, to preserve? What loses importance or priority? What will you let go of?

Day 3: Module 7 – Cultivating the spiritual dimension of life: A meaningful life

- MEDITATIONS
    1. Focusing the mind; centering and grounding; focusing on the breath.
    2. Reflecting on motivation; cultivating wholesome motivation for the day and for my life
    3. How we are all interconnected; recognizing the kindness of others; gratitude
    4. How letting go of self brings greater life satisfaction
- AFTERNOON TOPIC FOR REFLECTION AND DISCUSSION
    1. What am I taking away from my experience of this course? What things do I wish to continue to work on? How do I think to apply and live what I have learned in these last months? How do these things relate to making my life meaningful?
